# Minimum Information about an Uncultivated Virus Genome (MIUViG)

**DOI:** 10.1038/nbt.4306

**Published:** 2018-12-17

**Authors:** Simon Roux, Evelien M Adriaenssens, Bas E Dutilh, Eugene V Koonin, Andrew M Kropinski, Mart Krupovic, Jens H Kuhn, Rob Lavigne, J Rodney Brister, Arvind Varsani, Clara Amid, Ramy K Aziz, Seth R Bordenstein, Peer Bork, Mya Breitbart, Guy R Cochrane, Rebecca A Daly, Christelle Desnues, Melissa B Duhaime, Joanne B Emerson, François Enault, Jed A Fuhrman, Pascal Hingamp, Philip Hugenholtz, Bonnie L Hurwitz, Natalia N Ivanova, Jessica M Labonté, Kyung-Bum Lee, Rex R Malmstrom, Manuel Martinez-Garcia, Ilene Karsch Mizrachi, Hiroyuki Ogata, David Páez-Espino, Marie-Agnès Petit, Catherine Putonti, Thomas Rattei, Alejandro Reyes, Francisco Rodriguez-Valera, Karyna Rosario, Lynn Schriml, Frederik Schulz, Grieg F Steward, Matthew B Sullivan, Shinichi Sunagawa, Curtis A Suttle, Ben Temperton, Susannah G Tringe, Rebecca Vega Thurber, Nicole S Webster, Katrine L Whiteson, Steven W Wilhelm, K Eric Wommack, Tanja Woyke, Kelly C Wrighton, Pelin Yilmaz, Takashi Yoshida, Mark J Young, Natalya Yutin, Lisa Zeigler Allen, Nikos C Kyrpides, Emiley A Eloe-Fadrosh

**Affiliations:** 1 grid.451309.a 0000 0004 0449 479X US Department of Energy Joint Genome Institute, Walnut Creek, California USA; 2 grid.10025.36 0000 0004 1936 8470 Institute of Integrative Biology, University of Liverpool, Liverpool, UK; 3 grid.5477.1 0000000120346234 Theoretical Biology and Bioinformatics, Utrecht University, Utrecht, the Netherlands; 4 grid.10417.33 0000 0004 0444 9382 Centre for Molecular and Biomolecular Informatics, Radboud University Medical Centre, Nijmegen, the Netherlands; 5 grid.419234.9 0000 0004 0604 5429 National Center for Biotechnology Information, National Library of Medicine, National Institutes of Health, Bethesda, Maryland USA; 6 grid.34429.38 0000 0004 1936 8198 Department of Pathobiology, Ontario Veterinary College, University of Guelph, Guelph, Ontario Canada; 7 grid.428999.7 0000 0001 2353 6535 Institut Pasteur, Unité Biologie Moléculaire du Gène chez les Extrêmophiles, Paris, France; 8 Integrated Research Facility at Fort Detrick, National Institute of Allergy and Infectious Diseases, National Institutes of Health, Fort Detrick, Frederick, Maryland USA; 9 grid.5596.f 0000 0001 0668 7884 KU Leuven, Laboratory of Gene Technology, Heverlee, Belgium; 10 grid.215654.1 0000 0001 2151 2636 Biodesign Center for Fundamental and Applied Microbiomics, Center for Evolution and Medicine, School of Life Sciences, Arizona State University, Tempe, Arizona USA; 11 grid.7836.a 0000 0004 1937 1151 Department of Integrative Biomedical Sciences, Structural Biology Research Unit, University of Cape Town, Observatory, Cape Town, South Africa; 12 grid.225360.0 0000 0000 9709 7726 European Molecular Biology Laboratory, European Bioinformatics Institute (EMBL-EBI), Wellcome Genome Campus, Hinxton, UK; 13 grid.7776.1 0000 0004 0639 9286 Department of Microbiology and Immunology, Faculty of Pharmacy, Cairo University, Cairo, Egypt; 14 grid.152326.1 0000 0001 2264 7217 Departments of Biological Sciences and Pathology, Microbiology, and Immunology, Vanderbilt Institute for Infection, Immunology and Inflammation, Vanderbilt Genetics Institute, Vanderbilt University, Nashville, Tennessee USA; 15 grid.4709.a 0000 0004 0495 846X European Molecular Biology Laboratory, Heidelberg, Germany; 16 grid.170693.a 0000 0001 2353 285X College of Marine Science, University of South Florida, Saint Petersburg, Florida USA; 17 grid.47894.36 0000 0004 1936 8083 Soil and Crop Sciences Department, Colorado State University, Fort Collins, Colorado USA; 18 grid.5399.6 0000 0001 2176 4817 Aix-Marseille Université, CNRS, MEPHI, IHU Méditerranée Infection, Marseille, France; 19 grid.214458.e 0000000086837370 Department of Ecology & Evolutionary Biology, University of Michigan, Ann Arbor, Michigan USA; 20 grid.27860.3b 0000 0004 1936 9684 Department of Plant Pathology, University of California, Davis, Davis, California USA; 21 grid.462583.e 0000 0004 0385 0000 LMGE,UMR 6023 CNRS, Université Clermont Auvergne, Aubiére, France; 22 grid.42505.36 0000 0001 2156 6853 University of Southern California, Los Angeles, Los Angeles, California USA; 23 grid.5399.6 0000 0001 2176 4817 Aix Marseille Université, ,; 58 grid.4399.7 0000000122879528 , Université de Toulon, CNRS, IRD, MIO UM 110, Marseille, France; 24 grid.1003.2 0000 0000 9320 7537 Australian Centre for Ecogenomics, School of Chemistry and Molecular Biosciences, The University of Queensland, St. Lucia, Queensland Australia; 25 grid.134563.6 0000 0001 2168 186X Department of Agricultural and Biosystems Engineering, University of Arizona, Tucson, Arizona USA; 26 grid.134563.6 0000 0001 2168 186X BIO5 Research Institute, University of Arizona, Tucson, Arizona USA; 27 grid.264764.5 Department of Marine Biology, Texas A&M University at Galveston, Galveston, Texas USA; 28 grid.288127.6 0000 0004 0466 9350 DDBJ Center, National Institute of Genetics, Mishima, Shizuoka Japan; 29 grid.5268.9 0000 0001 2168 1800 Department of Physiology, Genetics and Microbiology, University of Alicante, Alicante, Spain; 30 grid.258799.8 0000 0004 0372 2033 Institute for Chemical Research, Kyoto University, Uji, Japan; 31 grid.417961.c Micalis Institute, INRA, AgroParisTech, Université Paris-Saclay, Jouy-en-Josas, France; 32 grid.164971.c 0000 0001 1089 6558 Department of Biology, Loyola University Chicago, Chicago, Illinois USA; 33 grid.164971.c 0000 0001 1089 6558 Bioinformatics Program, Loyola University Chicago, Chicago, Illinois USA; 34 grid.164971.c 0000 0001 1089 6558 Department of Computer Science, Loyola University Chicago, Chicago, Illinois USA; 35 grid.10420.37 0000 0001 2286 1424 Division of Computational Systems Biology, Department of Microbiology and Ecosystem Science, Research Network “Chemistry Meets Microbiology,” University of Vienna, Vienna, Austria; 36 grid.7247.6 0000000419370714 Department of Biological Sciences, Max Planck Tandem Group in Computational Biology, Universidad de los Andes, Bogotá, Colombia; 37 grid.26811.3c 0000 0001 0586 4893 Departamento de Producción Vegetal y Microbiología, Evolutionary Genomics Group, Universidad Miguel Hernández, Alicante, Spain; 38 grid.411024.2 0000 0001 2175 4264 University of Maryland School of Medicine, Baltimore, Maryland USA; 39 grid.410445.0 0000 0001 2188 0957 Department of Oceanography, Center for Microbial Oceanography: Research and Education, University of Hawai'i at Mānoa, Honolulu, Hawai'i USA; 40 grid.261331.4 0000 0001 2285 7943 Department of Microbiology, The Ohio State University, Columbus, Ohio USA; 41 grid.261331.4 0000 0001 2285 7943 Department of Civil, Environmental and Geodetic Engineering, The Ohio State University, Columbus, Ohio USA; 42 grid.5801.c 0000 0001 2156 2780 Department of Biology, ETH Zurich, Zurich, Switzerland; 43 grid.17091.3e 0000 0001 2288 9830 Department of Earth, Ocean and Atmospheric Sciences, University of British Columbia, Vancouver, British Columbia Canada; 44 grid.17091.3e 0000 0001 2288 9830 Department of Botany, University of British Columbia, Vancouver, British Columbia Canada; 45 grid.17091.3e 0000 0001 2288 9830 Department of Microbiology and Immunology, University of British Columbia, Vancouver, British Columbia Canada; 46 grid.17091.3e 0000 0001 2288 9830 Institute of Oceans and Fisheries, University of British Columbia, Vancouver, British Columbia Canada; 47 grid.8391.3 0000 0004 1936 8024 School of Biosciences, University of Exeter, Exeter, UK; 48 grid.4391.f 0000 0001 2112 1969 Department of Microbiology, Oregon State University, Oregon, USA., ,; 49 grid.1046.3 0000 0001 0328 1619 Australian Institute of Marine Science, Townsville, Queensland Australia; 50 grid.266093.8 0000 0001 0668 7243 Department of Molecular Biology and Biochemistry, University of California, Irvine, California USA; 51 grid.411461.7 0000 0001 2315 1184 Department of Microbiology, University of Tennessee, Knoxville, Tennessee USA; 52 grid.33489.35 0000 0001 0454 4791 University of Delaware, Delaware Biotechnology Institute, Newark, Delaware USA; 53 grid.419529.2 0000 0004 0491 3210 Microbial Physiology Group, Max Planck Institute for Marine Microbiology, Bremen, Germany; 54 grid.258799.8 0000 0004 0372 2033 Graduate School of Agriculture, Kyoto University, Kitashirakawa-Oiwake, Kyoto, Japan; 55 grid.41891.35 0000 0001 2156 6108 Department of Plant Sciences and Plant Pathology, Montana State University, Bozeman, Montana USA; 56 grid.469946.0 J Craig Venter Institute, La Jolla, California USA; 57 grid.266100.3 0000 0001 2107 4242 Scripps Institution of Oceanography, University of California, San Diego, La Jolla, California, USA., ,

**Keywords:** Environmental microbiology, Phage biology, Genetic databases, Virology, Metagenomics

## Abstract

We present an extension of the Minimum Information about any (x) Sequence (MIxS) standard for reporting sequences of uncultivated virus genomes. Minimum Information about an Uncultivated Virus Genome (MIUViG) standards were developed within the Genomic Standards Consortium framework and include virus origin, genome quality, genome annotation, taxonomic classification, biogeographic distribution and *in silico* host prediction. Community-wide adoption of MIUViG standards, which complement the Minimum Information about a Single Amplified Genome (MISAG) and Metagenome-Assembled Genome (MIMAG) standards for uncultivated bacteria and archaea, will improve the reporting of uncultivated virus genomes in public databases. In turn, this should enable more robust comparative studies and a systematic exploration of the global virosphere.

**Supplementary information:**

The online version of this article (doi:10.1038/nbt.4306) contains supplementary material, which is available to authorized users.

## Abstract (web summary)

This paper presents standards and best practices for reporting genome sequences of uncultivated viruses.

**Supplementary information:**

The online version of this article (doi:10.1038/nbt.4306) contains supplementary material, which is available to authorized users.

## Main

**Figure 1:**
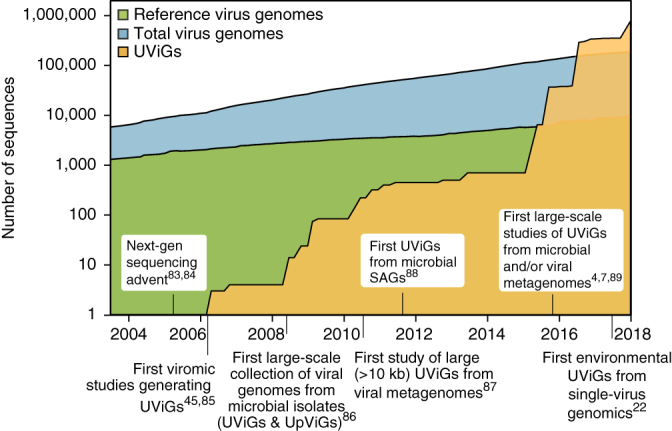
Size of virus genome databases over time^4,7,22,45,83,84,85,86,87,88,89^. Genome sequences from isolates (blue and green) or from UViGs (yellow) are shown. For genomes from isolates, the total number of genomes (blue) and the number of 'reference' genomes (green) are shown. Data were downloaded using the queries “Viruses[Organism] AND srcdb_refseq[PROP] NOT wgs[PROP] NOT cellular organisms[ORGN] NOT AC_000001:AC_999999[PACC]” for reference genomes and “Viruses[Organism] NOT cellular organisms[ORGN] NOT wgs[PROP] NOT AC_000001:AC_999999[pacc] NOT gbdiv syn[prop] AND nuccore genome samespecies[Filter]” for total number of virus genomes, on the NCBI nucleotide database portal (https://www.ncbi.nlm.nih.gov/nuccore) in January 2018. Genomes from the influenza virus database (https://www.ncbi.nlm.nih.gov/genomes/FLU/Database/nph-select.cgi?go=genomeset) were also added to the total number of virus genomes. UViGs can be assembled from metagenomes, from proviruses identified in microbial genomes, or from single-virus genomes, and estimated total UViG numbers were obtained by compiling data from the literature and from the total number of sequences in the IMG/VR database in January 2017, January 2018 and July 2018 (https://img.jgi.doe.gov/vr/)^11^. UpViG, uncultivated provirus.

Current estimates are that virus particles massively outnumber live cells in most habitats^1,2^, but only a tiny fraction of viruses have been cultivated in the laboratory. An unprecedented diversity of viruses are being discovered through culture-independent sequencing^3^. Progress has been made in reconstructing genomes of uncultivated viruses *de novo*, from biotic and abiotic environments, without laboratory isolation of the virus–host system. For example, in the past 2 years, more than 750,000 uncultivated virus genomes (UViGs) have been identified in metagenome and metatranscriptome datasets^4,5,6,7,8,9^, five times the total number of genomes sequenced from virus isolates (Fig. 1), and UViGs already represent ≥95% of the taxonomic diversity in publicly available virus sequences^10,11^. Although double-stranded DNA (dsDNA) genomes are over-represented in UViGs because most metagenomic protocols exclusively target dsDNA, UViGs nonetheless enable an assessment of global virus diversity and an evaluation of structure and drivers of viral communities. UViGs also contribute to improving our understanding of the evolutionary history of viruses and virus–host interactions.

Analysis and interpretation of standalone genomes present substantial challenges, whether the genomes are eukaryotic, bacterial, archaeal or viral. To address these challenges, MISAG and MIMAG standards were drafted to improve the quality of reporting of microbial genomes derived from single cell or metagenome sequences, which are often incomplete^12^. Although some aspects of MISAG and MIMAG can be applied to UViGs, the extraordinary diversity of viral genome composition and content, replication strategies, and hosts means that the completeness, quality, taxonomy and ecology of UViGs need to be evaluated via virus-specific metrics.

The Genomic Standards Consortium (http://gensc.org) maintains metadata checklists for MIxS, encompassing genome and metagenome sequences^13^, marker gene sequences^14^ and single amplified and metagenome-assembled bacterial and archaeal genomes^12^. Here we present a set of standards that extend the MIxS checklists to include identification, quality assessment, analysis and reporting of UViGs (Table 1 and Supplementary Tables 1 and 2), together with recommendations on how to perform these analyses. We provide a metadata checklist for database submission and publication of UViGs designed to be flexible enough to accommodate technological and methodological changes over time (Table 1 and Supplementary Table 1). The information gathered through the MIUViG checklist can be directly submitted with new UViG sequences to International Nucleotide Sequence Database Collaboration (INSDC) member databases—the DNA Database of Japan (DDBJ), the European Molecular Biology Laboratory–European Bioinformatics Institute (EMBL-EBI) and US National Center for Biotechnology Information (NCBI)—which will host and display checklist metadata alongside the UViG sequence. These MIUViG standards should also be used along with existing guidelines for virus genome analysis, including those issued by the International Committee on Taxonomy of Viruses (ICTV), which recently endorsed the incorporation of UViGs into the official virus classification scheme^15^ (https://talk.ictvonline.org). Although MIUViG standards and best practices were designed for genomes of viruses infecting microorganisms, they can also be applied to viruses infecting animals, fungi and plants, and are compatible with standards that are already in place for epidemiological analysis of these viruses^16^ (Supplementary Table 3).

**Table 1:** List of mandatory metadata for UViGs

Mandatory metadata	Description
Source of UViGs	Type of dataset from which the UViG was obtained
Assembly software	Tool(s) used for assembly and/or binning, including version number and parameters
Virus identification software	Tool(s) used for the identification of UViG as a viral genome, software or protocol name including version number, parameters, and cutoffs used (see Supplementary Table 2)
Predicted genome type	Type of genome predicted for the UViG
Predicted genome structure	Expected structure of the viral genome
Detection type	Type of UViG detection
Assembly quality	The assembly quality categories, specific for virus genomes, are based on sets of criteria as follows: **Finished:** Single, validated, contiguous sequence per replicon without gaps or ambiguities, with extensive manual review and editing to annotate putative gene functions and transcriptional units **High-quality draft genome:** One or multiple fragments, totaling ≥90% of the expected genome or replicon sequence or predicted complete **Genome fragment(s):** One or multiple fragments, totaling <90% of the expected genome or replicon sequence, or for which no genome size could be estimated
Number of contigs	Total number of contigs composing the UViG

For a complete list and description of mandatory and optional metadata, see Supplementary Table 1.

### Recovery of UViGs after virus enrichment

**Figure 2:**
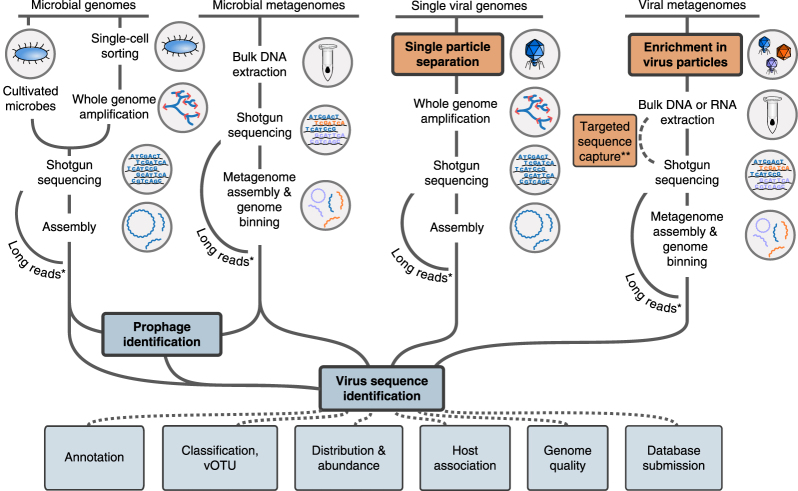
Identification of UViGs. Schematic of methods used to obtain UViGs. Steps that have been adapted from those used to assemble MAGs and SAGs^12^ or added for UViG are shown for sample preparation (orange) and bioinformatics analysis (blue). Steps specifically required for virus targeting and identification are highlighted in bold. *For viruses with short genomes, long-read technologies can provide complete genomes from shotgun sequencing in a single read, bypassing the assembly step^24^. **Targeted sequence capture can be used to recover viral genomes from a known virus group. These genomes can be recovered from samples in which they represent a small fraction of the templates (for example, clinical samples^20^).

UViGs can be retrieved from datasets enriched for virus genomes, namely viral metagenomes and single-virus genomes (Fig. 2). Viral metagenomes are usually obtained through a combination of filtration steps, DNase or RNase treatments, and RNA or DNA extraction depending on the targeted viruses, then reverse transcription (to find RNA viruses) and shotgun sequencing^3,17,18,19^. Targeted sequence capture methods can be applied to recover specific virus groups (Fig. 2), and these methods have proven especially useful when viruses are present in small amounts (for example, clinical samples)^20^. Single-virus methods use flow cytometry to sort individual viral particles before genome amplification and sequencing, to produce viral single amplified genomes (SAGs)^9,21,22,23^ (Fig. 2). Viral metagenomes and single-virus genomes are usually sequenced with short-read, high-throughput technologies, such as Illumina sequencing, and assembled by algorithms similar to those used for microbial genomes and metagenomes. However, owing to their relatively small genome size (92% of virus genomes in the NCBI Viral RefSeq database are <100 kb)^10^, short read-based genome assemblies could soon be superseded by long-read sequencing technologies^24^ (for example, PacBio zero-mode waveguide technology or Oxford Nanopore Technology nanopore sequencing; Fig. 2). Sequencing virus genomes from a single template would notably enable the identification of individual genotypes in mixed populations.

The main advantages of datasets produced after enrichment for viruses are good *de novo* assembly of both abundant and rare viruses, increased confidence that the sequence is of viral origin, and the ability to sequence both active and 'inactive' or 'cryptic' viruses (i.e., viruses that are present in the sample but cannot infect). However, virus-enriched datasets can have over-representation of virulent viruses with high burst size (high number of virus particles released from each infected cell) and under-representation of larger viruses with capsids ≥0.2 μm, such as giant viruses, as a result of the selective filtration steps used^25^. Furthermore, *in silico* approaches are often the only option available to determine the host range of UViGs obtained from virus-enriched samples.

### Recovery of UViGs without enrichment

Virus sequences are also present in non-virus-enriched datasets, including sorted cells, tissues, or environmental samples collected on 0.2 μm filters^4,26,27,28^. These sequences could originate from viruses that are replicating in cells, from temperate viruses (proviruses or prophages) that are either integrated into host genomes or present as episomal elements in the host cell, or from free virus particles present in samples.

Analyzing datasets without virus enrichment has several advantages. It can detect lytic, temperate and persistent infection, it overcomes some of the biases arising from the size-based selection of virus particles, and it can be applied to any metagenome. However, UViGs from non-virus-enriched datasets may be biased toward viruses that infect the dominant host cell in the sample, and rare viruses or those infecting rare hosts could be under-represented or absent. Finally, comparisons between virus-enriched and non-virus-enriched datasets suggest that analyzing UViGs across different size fractions and sample types is valuable for exploring the virus genome sequence space^29^ (Supplementary Fig. 1 and Supplementary Note 1).

### Computational identification of viral sequences

Regardless of the type of dataset, the viral origin of UViGs must be validated because even samples enriched for virus particles still contain a substantial amount of cellular DNA^30^. Contamination can arise either from difficulty in separating virus particles from cellular fractions (for example, ultra-small bacteria^31^) or from the capture of extracellular DNA in the virus fraction. Cellular sequences can also derive from cell genome fragments that are encased in virus capsids or comparable particles (for example, via transduction), DNA-containing membrane vesicles, or gene transfer agents^32,33,34^.

Several bioinformatic tools and protocols have been developed to identify sequences from bacteriophages and archaeal viruses^35,36,37,38^; eukaryotic viruses^39^; or combinations of bacteriophages, archaeal viruses and large eukaryotic viruses^40^ (Supplementary Table 4). These approaches rely on a few characteristics, such that a sequence is considered viral if it is significantly similar to known viruses (in terms of gene content or nucleotide usage pattern) or if it is unrelated to any known virus and cellular genome but contains one or more hallmark virus genes. UViGs must therefore be accompanied by a list of virus detection tool(s) and protocol(s) used, together with any thresholds applied (Table 1 and Supplementary Table 1).

Identification of integrated proviruses and their precise boundaries in the host genome is problematic (Box 1). Notably, no high-throughput approach can accurately distinguish active proviruses (still able to replicate and produce virions) from inactive proviral remnants of a past infection^28^. Thus, although prediction methods are improving, UViGs identified as proviruses should be clearly marked as such, so that these caveats are clear (Table 1 and Supplementary Table 1).

Box 1: Problems and pitfalls in assembly of uncultivated virus genomes Several factors may confound assembly of an uncultivated virus genome. The major issues are listed below:

• **Misidentification of a cellular sequence as viral.** Viral metagenomes can be contaminated with cellular nucleic acids^30^. Any analysis should start with the identification of virus and cellular sequences, even in virus-targeted datasets. We advise process improvement by analyzing replicates, blanks or other controls. Determining the boundaries of an integrated provirus can be challenging, even for dedicated software (for example, PHAST, VirSorter), which can results in inclusion of host gene(s) in a virus genome. Manual annotation of genes on the edge of a provirus prediction is recommended.

• **Partial genomes assembled as circular contigs.** Partial genomes are sometimes misassembled as circular contigs owing to repeats^47^. These circularized fragments could be incorrectly identified as complete genomes. The size and gene content of circular contigs should be manually validated as consistent or at least plausible in comparison with known reference genomes.

• **Errors in gene prediction.** For novel viruses with little or no similarity to known references, gene prediction can be challenging in the absence of accompanying transcriptomics or proteomics data. Outputs of automatic gene predictors applied to novel viruses should be checked for gene density (most viruses do not include large noncoding regions), as well as typical gene prediction errors, such as internal stop codons causing artificially shortened genes.

• **Inaccurate functional annotation.** The annotation of open reading frames predicted from novel viruses often requires sensitive profile similarity approaches. Although such sensitive searches are necessary to detect homology in the face of high rates of virus sequence evolution, the inferred function should be cautiously interpreted and remain general (for example, “DNA polymerase,” “membrane transporter” or “PhoH-like protein”).

• **Clustering of partial genomes.** Incomplete genomes can be difficult to classify using genome-based taxonomic classification methods. For example, the estimation of whole-genome average nucleotide identity from partial genomes could vary by up to 50% from the complete genome value (Supplementary Fig. 5). Thus, the classification of genome fragments and their clustering into vOTUs should be interpreted only as an approximation of the true clustering values, and it will likely change as more complete genomes become available.

• **Taxonomic classification of UViG.** Although virus classification primarily relies on genome sequences, no universal approach is currently available to classify viruses at different ranks. Classification of UViGs should be based on the best method available for the type of virus (see Box 2).

• **Read mapping from nonquantitative datasets**. Amplified datasets, produced using multiple displacement amplification or sequence-independent single-primer amplification, are biased toward specific virus genome types and can selectively overamplify specific genome regions. The coverage derived from read mapping based on these amplified datasets should not be interpreted as reflecting the relative abundance of the UViG in the initial sample.

### Estimating quality of UViGs

**Figure 3:**
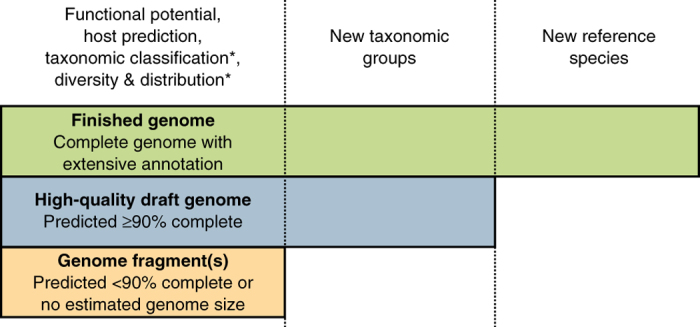
UViG classification and associated sequence analyses. “Functional potential” is functional annotation used in gene content analysis. “Host prediction” is the application of different *in silico* host prediction tools. “Taxonomic classification” is classification of the contig to established groups using marker genes or gene content comparison. “Diversity and distribution” includes vOTU clustering and relative abundance estimation through metagenome read mapping, at the geographical scale or across anatomical sites for host-associated datasets. “New taxonomic groups” concerns the delineation of new proposed groups (for example, families or genera) based exclusively on UViG sequences. “New reference species” refers to the proposal of a new entry in ICTV (https://talk.ictvonline.org/files/taxonomy-proposal-templates/). *Some of these approaches require a minimum contig size—for example, contigs ≥10 kb for taxonomic classification based on gene content^59^ or diversity estimation^47^—and will not be applicable to every genome fragment.

We propose three categories of UViG sequences: genome fragment(s), high-quality draft genomes and finished genomes (Fig. 3 and Table 2). These categories mirror those in MISAG and MIMAG^12^, and they are matched to categories already proposed for complete-genome sequencing of small viruses in epidemiology and surveillance^16^ (Supplementary Table 3). UViG quality is more challenging to evaluate than metagenome-assembled genomes (MAGs) or SAGs because most viruses lack conserved sets of single-copy marker genes that can be used to estimate draft genome completeness. However, exceptions exist, such as large eukaryotic dsDNA viruses. To date, researchers have estimated UViG sequence completeness by identifying circular contigs or contigs with inverted terminal repeats as putative complete genomes. For linear contigs, completeness is estimated by comparison to reference genome sequences and typically requires a taxonomic assignment to a (candidate) (sub)family or genus because genome length is relatively homogeneous at these ranks (±10%; Supplementary Fig. 2 and Supplementary Table 5). This assignment can be based on the detection of specific marker genes, such as clade-specific viral orthologous groups (Supplementary Table 6), or based on genome-based classification tools (see “Taxonomy of UViGs”). Estimating completeness is more difficult for segmented genomes, which require either a closely related reference genome or additional *in vitro* experiments^16^. A detailed example of how this quality tier classification can be performed on the Global Ocean Virome dataset^7^ is presented in Supplementary Note 2 and Supplementary Table 7.

**Table 2:** Summary of required characteristics for each category

Category	Genome fragment(s)	High-quality draft genome	Finished genome
Assembly	Single or multiple fragments	Single or multiple fragments where gaps span (mostly) repetitive regions	Single contiguous sequence (per segment) without gaps or ambiguities
Completeness	<90% expected genome size or no expected genome size	Complete or ≥90% of expected genome size	Complete
Required features	Minimal annotation	Minimal annotation	Comprehensive manual review and editing

Complete genomes include sequences detected as circular, those with terminal inverted repeats, or those for which an integration site is identified.

Contigs or genome bins representing <90% of the expected genome length, or for which no expected genome length can be determined, would be considered genome fragments. This category might include UViG fragments large enough to be assigned to known virus groups on the basis of gene content and average nucleotide identity. However, high-quality draft or finished genomes are required to establish new taxa (Fig. 3). Sequences from UViG fragments can be used in phylogenetic and diversity studies, either as references for virus operational taxonomic units (see Supplementary Note 4), or through the analysis of virus marker genes encoded in these genome fragments; for example, capsid proteins, terminases, ribonucleotide reductases and DNA- or RNA-dependent RNA polymerases^41,42,43,44,45,46^. Similarly, UViG fragments can be analyzed to assess the functional gene complement of unknown viruses or link them to potential hosts. Importantly, current methods for automatic virus sequence identification^35,36,37,38,39,40^ cannot reliably identify short (<10 kb) viral sequences, which should be interpreted with utmost caution.

Contigs or genome bins either predicted as complete or representing ≥90% of the expected genome sequence are high-quality drafts, consistent with standards for microbial genomes^12^. Repeat regions may lead to erroneous assembly of partial genomes as circular contigs^47^. Thus, the length of the assembled circular contig should be considered when assessing UViG completeness (Box 1). For UViGs not derived from a consensus assembly, such as single long reads, base calling quality >99% on average (phred score >20) is needed to assign a “high-quality draft” label. Genome sequences assembled into a single contig, or one per segment, with extensive manual review and annotation, can be labeled “finished genomes.” Annotation must include identification of putative gene functions; structural, replication or lysogeny modules; and transcriptional units. The “finished genomes” category is reserved for only the highest quality, manually curated UViGs and is required for the establishment of new virus species (Fig. 3 and Table 2).

Unlike that of SAGs and MAGs^12^, quality estimation of UViGs does not include a genome contamination threshold. Contamination issues are most prominent in the case of genome bins, whereas most UViGs are represented by a single contig for which *in silico* simulations have shown that chimeric sequences are rare and present at <2% (ref. 47). In addition, no tools exist to automatically estimate UViG contamination, and thus this information is not included in the current MIUViG checklist. A future updated version of the MIUViG checklist may, however. For include contamination thresholds if such a tool were to be developed. For example, such a tool might exploit single-copy marker genes (once these have been defined for a broader range of viruses) or it might use coverage by metagenome reads, which should in principle be evenly distributed along the genome with no major deviance, except for highly conserved genes.

### Annotation of UViGs

Functional annotation of UViGs comprises the following tasks: predicting features in the genome sequence, such as protein-coding genes, tRNAs and integration sites; assigning functions to as many predicted features as possible; and assigning the remaining hypothetical proteins to uncharacterized protein families. Annotation pipelines have been established for different types of viruses^48,49^, and large differences between viral genome types likely preclude the development of a single tool able to annotate every virus^50^. Therefore, we recommend that software used to annotate UViGs be reported (Supplementary Table 1).

The choice of methods and reference databases used to annotate predicted proteins should be clearly stated. Homologs of novel virus genes may not be detected with standard methods for pairwise sequence similarity detection, such as BLAST, but instead require the use of more sensitive profile similarity approaches, such as HMMER^51^, PSI-BLAST^52^ or HHPred^53^ (Supplementary Table 8; reviewed in ref. 54). Although sequence profiles for many protein families have been collected, they frequently remain unassociated with any specific function. Therefore, UViG analyses should always report (i) feature prediction method(s), (ii) sequence similarity search method(s), and (iii) database(s) searched (Box 1 and Supplementary Table 1).

### Taxonomy of UViGs

Taxonomic classification can provide information on the relationship of a UViG with known viruses. Although the information and criteria used for virus classification have changed over time, virus classification has now converged to genome-based analyses^15^ (Box 2). The ICTV established specific demarcation criteria for each virus group (Supplementary Table 9) owing to the vast range of viral genomes, mutation rates and evolution. Recently, a consensus has emerged on using whole-genome average nucleotide identity for classification at the species rank, which is used in downstream ecological, evolutionary and functional studies. This consensus was reached through analysis of published population genetics studies^55,56^ and gene content comparison of NCBI RefSeq^10^ virus genomes^57,58,59^ (Supplementary Note 3 and Supplementary Fig. 3). We propose to formalize the use of species-rank virus groups and to name these “virus operational taxonomic units” (vOTUs) to avoid confusion because species groups have been variously named “viral population,” “viral cluster” or “contig cluster” in the literature^4,7,60^. We suggest standard thresholds of 95% average nucleotide identity over 85% alignment fraction (relative to the shorter sequence) on the basis of a comparison of sequences currently available in NCBI RefSeq^10^ and IMG/VR^11^ (Supplementary Note 3 and Supplementary Figs. 3 and 4). Although partial genomes remain challenging to classify, these common thresholds will enable comparative analyses (Supplementary Fig. 5). In addition, vOTU reports should include the clustering method and cutoff, the reference database used (if any), and the genome alignment approach because small differences have been observed between different methods^61^ (Supplementary Table 1).

For higher taxonomic ranks than species, no consensus has been reached on which approach should be used, although several have been proposed^58,59,62,63,64,65,66^. Keeping this in mind, UViG reports including taxonomy must clearly indicate the methods and cutoffs applied, and any new taxon must be highlighted as preliminary (for example, “genus-rank cluster,” “putative genus” or “candidate genus,” but not simply “genus,” as this category is reserved for ICTV-recognized groups; Supplementary Table 1). Authors should submit formal taxonomic proposals to the ICTV for consideration (https://talk.ictvonline.org/files/taxonomy-proposal-templates/).

Finally, information about the nature of the genome and mode of expression (i.e., Baltimore classification^67^) should be included in the UViG description. Similarly, the predicted segmentation state of the genome (segmented or nonsegmented) should be reported, typically derived from taxonomic classification and comparison with the closest references (Supplementary Table 1).

Box 2: Virus taxonomy Compared with the classification of cellular organisms, virus classification is associated with unique challenges. First, viruses are most likely polyphyletic; that is, they arose multiple times independently. Unlike ribosomal genes of cellular organisms, for example, there are no genes that are present in all virus genomes that could be used as universal taxonomic markers. Virus genomes are variable, and they can be single-stranded RNA (or single-stranded DNA) encoding only a couple of proteins, double-stranded RNA viruses with up to 12 segments, or large and complex dsDNA viruses with genome sizes that are as large as those of some bacteria. Viruses are very diverse and tend to evolve faster than cellular organisms, in terms of both their genetic sequence and genome content. For all these reasons, viruses are not incorporated into the universal tree of life and a 'one size fits all' virus taxonomy has not been reported. Instead, there are different classification rules for different groups of viruses.

A set of criteria to classify viruses was first formally proposed by the Virus Subcommittee of the International Nomenclature Committee at the Fifth International Congress of Microbiology, held at Rio de Janeiro in August 1950 (ref. 90). The virus classification criteria were purposefully based on stable properties of the virus itself, first among them being the virion morphology, virus genome type, and mode of replication, rather than more variable properties such as symptomatology after infection. A hierarchical categorization of viruses based on genome type and virion morphology was then proposed^91^, and another operational classification scheme relying on nucleic acid type and method of genome expression was proposed by David Baltimore in 1971 (ref. 67).

The need for a specific set of rules to name and classify viruses led to the establishment of the International Committee on Nomenclature of Viruses (ICNV)^92^, renamed as the International Committee on Taxonomy of Viruses (ICTV) in 1975 (ref. 82). The ICTV is a committee of the Virology Division of the International Union of Microbiological Societies and is charged with the task of developing, refining and maintaining the official virus taxonomy, presented to the research community in *The ICTV Report* (https://talk.ictvonline.org/ictv-reports/ictv_online_report/) and interim update articles (“Virology Division news”) in *Archives of Virology*. Using some of the stable properties of viruses that were previously highlighted, experts in the ICTV developed a universal virus taxonomy similar to the classical Linnaean hierarchical system, in which virus groups were assigned to familiar taxonomic ranks including order, family, genus and species.

In the postgenomic era, virus classification is increasingly based on the comparison of genome and protein sequences, which provides a unique opportunity to evaluate phylogenetic and evolutionary relationships between viruses and reconcile the taxonomy of viruses with their reconstructed evolutionary trajectory. The ICTV has undertaken the immense task of re-evaluating virus classification in light of sequence-based information^15,82,93^. Importantly, with large sections of the virosphere still to be explored, virus taxonomy represents only the current best attempt at recapitulating virus evolutionary history on the basis of available data. Virus classification will need to remain dynamic, expanding as we discover new viruses and being refined as our understanding of virus evolution improves.

### *In silico* host prediction

Once a new virus genome has been assembled, an important step toward understanding the ecological role of the associated virus is to predict its host(s). *In silico* approaches are often the only option for UViGs (reviewed in ref. 68; Supplementary Table 10). These can be separated into four main types. First, hosts can be predicted with relatively high precision on the basis of sequence similarity between the UViG and a reference virus genome when a closely related virus is available^69,70^. Second, hosts can be predicted on the basis of sequence similarities between a UViG and a host genome. These sequence similarities can range from short exact matches (∼20–100 bp), which include CRISPR spacers^4,7,68,71^, to longer (>100 bp) nucleotide sequence matches, including proviruses integrated into a larger host contig^26,68,72,73^ (Supplementary Table 10). Host-range predictions based on sequence similarity are the most reliable but require that a closely related host genome has been sequenced^68^. Third, host taxonomy from domain down to genus rank can be predicted from nucleotide usage signatures reflecting coevolution between virus and host genomes in terms of G+C content, *k*-mer frequency and codon usage^26,74,75^. These approaches are usually less specific than sequence similarity–based ones and cannot reliably predict host range below the genus rank, but can provide a predicted host for a larger number of UViGs^7^ (Supplementary Table 10). Finally, host predictions can be computed from a comparison of abundance profiles of host and virus sequences across spatial or temporal scales, either through abundance correlation^25,76,77,78^ or through more sophisticated model-based interaction predictors^79^. Although few datasets are available for robust evaluation of host prediction based on comparison of abundance profiles, we expect this approach to become more powerful and relevant as high-resolution time-series metagenomics becomes more common.

As all these bioinformatic approaches remain predictive, it is crucial that robust false-discovery rate estimations are reported (Supplementary Table 1). Moreover, computational tools do not predict quantitative infection characteristics (for example, infection rate or burst size), which are important for understanding the impacts of viruses on host biology, and thus far only apply to viruses infecting bacteria or archaea. Nevertheless, these predictions are important guides for subsequent *in silico*, *in vitro* and *in vivo* studies, including experimental validation to unequivocally demonstrate a viral infection of a given microbial host. Host predictions should be reported along with details regarding the specific tool(s) used and, importantly, their estimated accuracy as derived either from published benchmarks or from tests conducted in the study (Supplementary Table 1). This information will allow virus–host databases^69,80^ to progressively incorporate UViGs while still controlling for the sensitivity and accuracy of the predictions provided to users.

### Reporting UViGs

We recommend the following best practice for sharing and archiving UViGs and UViG-related data: data publication should center on the data resources of INSDC (http://www.insdc.org/) through one of the member databases, at DDBJ (https://www.ddbj.nig.ac.jp/index-e.html), EMBL-EBI's European Nucleotide Archive (ENA; https://www.ebi.ac.uk/ena) or NCBI (GenBank and the Sequence Read Archive; https://www.ncbi.nlm.nih.gov/nucleotide). If needed, INSDC database curators can be contacted directly for large-scale batch dataset submissions. Where new datasets are generated as part of a UViG study, sequenced samples should be described according to the environment-relevant MIxS checklists and raw read data should be submitted. High-quality and finished UViGs should be submitted as assemblies, the former reported as “draft” accompanied by the required metadata (Table 1). Incomplete assemblies may be submitted, but they must be accompanied by the required metadata (Table 1 and Supplementary Table 1).

Where available, annotation and taxonomic classification should be submitted to INSDC, and occurrence and abundance data reported as 'Analysis' records in the ENA. Reports of abundance data estimated by short-read metagenome mapping should include information about the nucleotide identity and coverage thresholds used, with corresponding estimates of false-positive and false-negative rates either computed *de novo* or extracted from the literature (for example, from refs. 47, 81; Supplementary Note 4). All INSDC accession codes must be cited in publications. For ICTV classification, only coding-complete genomes (complete high-quality and finished draft UViGs) are currently considered^82^.

### Conclusions

MIUViG standards and best practices for UViG analysis are the virus-specific counterparts to MISAG and MIMAG^12^. Virus genomics and metagenomics are rapidly expanding and improving as sequencing technologies emerge and mature. At the same time, the development of genome-based virus taxonomy methods as well as unified, comprehensive, and annotated reference databases of virus genomes and/or proteins continues apace. Community adoption of these standards, including through ongoing collaborations with other virus committees (ICTV) and data centers (DDBJ, EMBL-EBI and NCBI), will provide a framework for a systematic exploration of viral genome sequence space and enable the research community to better utilize and report UViGs.

## Additional information

**Publisher's note:** Springer Nature remains neutral with regard to jurisdictional claims in published maps and institutional affiliations.

## Supplementary Information

### Integrated supplementary information

Supplementary Figure 1 Comparison of UViG recovery from microbial (“M”) and viral (“V”) metagenomes originating from the same *Tara* Oceans samples. Top panel represents the number of distinct virus contigs ≥ 10kb identified in each dataset. The bottom panel depicts the ratio of “shared”, i.e., detected in both viral and microbial fraction of the sample, and “unique”, i.e., detected only in one fraction, contigs in each microbial and viral fraction. Datasets were originally analyzed in refs. ^1,2^. SRF: surface, DCM: deep chlorophyll maximum.

Supplementary Figure 2 Genome size variation for different types of viruses and different taxonomic levels. Genome length of virus genomes from NCBI RefSeq were compared at different taxonomic ranks and are presented separately for four main types of viruses (dsDNA, ssDNA, RNA and reverse-transcribing RNA, viroids and satellites). Genome length variation was calculated as a coefficient of variation, i.e. standard deviation of genome length in the group divided by average genome length in the grouop (for groups with >1 genome). Underlying data are available in Supplementary Table 5. Boxplots lower and upper hinges correspond to the first and third quartiles (the 25th and 75th percentiles), while whisker extend from the nearest hinge to the smallest/largest value no further than 1.5 * IQR from the hinge (where IQR is the inter-quartile range, or distance between the first and third quartiles). dsDNA: double-stranded DNA; ssDNA: single-stranded DNA.

Supplementary Figure 3 Pairwise average nucleotide identity (ANI) and alignment fraction (AF) for NCBI Viral RefSeq genomes and IMG/VR. Only genome pairs with ANI >60% and AF >20% were considered. ANI and AF were binned in 1% intervals, and are represented here as a heatmap (i.e. cell coloring represents the number of pairwise comparisons at the corresponding ANI and AF intervals). On the top right corner (i.e., AF and ANI close to 100%), three main groups of genome pairs are delineated with black dashed circles, and the proposed standard cutoff is highlighted in dark red. Note that for this clustering, the cutoff was applied as follows: pairs of genomes with ≥ 85% AF were first selected, and whole genome (wg) ANI was then calculated by multiplying the observed ANI by the observed AF. This wgANI was then compared to the corresponding whole genome ANI cutoff (i.e. 95% ANI * 85% AF = 80.75% wgANI). This allows for hits with ≤ 95% ANI but ≥ 85 % AF to be considered as well, i.e. a pair of genomes with 90% ANI on 100% AF would be considered as “passing” the cutoff. Examples of genome comparisons for each group are presented in Supplementary Fig. 4.

Supplementary Figure 4 Examples of pairwise genome comparisons from the three groups of genome pairs highlighted in Supplementary Figure 3. For each example, nucleotide similarity (blastn) and amino acid similarity (tblastx) are displayed, alongside the ANI, AF, and wgANI (i.e. ANI over the whole length of the shorter genome). AF, alignment fraction; ANI, average nucleotide identity; wgANI, whole-genome average nucleotide identity.

Supplementary Figure 5 Estimation of whole genome ANI from fragmented genomes. To evaluate the impact of genome fragmentation on whole-genome average nucleotide identity (wgANI) estimation, pairs of genomes from NCBI RefSeq with wgANI ≥ 70% and ≥ 20kb were selected, random fragments were generated (from 1 to 45kb) from one of the two genomes, and then compared to the other complete genome. The resulting estimated wgANI between the fragment and complete genome was then compared with the original values estimated from the two complete genomes (y-axis). Boxplots lower and upper hinges correspond to the first and third quartiles (the 25th and 75th percentiles), while whisker extend from the nearest hinge to the smallest/largest value no further than 1.5 * IQR from the hinge (where IQR is the inter-quartile range, or distance between the first and third quartiles).

### Supplementary information

Supplementary Text and Figures Supplementary Figures 1–5 (PDF 1122 kb)

Supplementary Notes Supplementary Notes 1–4 (PDF 196 kb)

Supplementary Table 1 List of mandatory and optional metadata for UViGs (XLSX 9 kb)

Supplementary Table 2 List of metadata from previous standards relevant for UViGs^21^ (XLSX 17 kb)

Supplementary Table 3 Comparison between UViGs categories and the quality categories proposed for small DNA/RNA virus whole-genome sequencing for epidemiology and surveillance by Ladner *et al*.^22^ (XLSX 5 kb)

Supplementary Table 4 List and characteristics of tools used to identify virus sequences in mixed datasets published or updated since 2012^23–31^ (XLSX 6 kb)

Supplementary Table 5 Variation in genome length for virus families and genera with two or more genomes, from NCBI RefSeq v83. (XLSX 25 kb)

Supplementary Table 6 List of potential marker genes for virus orders, families or genera, based on the VOGdb v83 (http://vogdb.org/) (XLSX 85 kb)

Supplementary Table 7 List of UViGs from the GOV dataset^4^ considered as high-quality drafts or finished genomes (XLSX 38 kb)

Supplementary Table 8 List of databases providing collections of HMM profiles for virus protein families^32–35^ (XLSX 6 kb)

Supplementary Table 9 Current species demarcation criteria from ICTV ninth and tenth reports. (XLSX 46 kb)

Supplementary Table 10 Approaches available for *in silico* host prediction^18,37–42^ (XLSX 6 kb)

## References

[1] Breitbart M, Bonnain C, Malki K, Sawaya NA (2018). Phage puppet masters of the marine microbial realm. Nat. Microbiol..

[2] Youle, M., Haynes, M. & Rohwer, F. in *Viruses: Essential Agents of Life* (ed. Witzany, G.) 61–81 (Springer Netherlands, 2012).

[3] Brum JR, Sullivan MB (2015). Rising to the challenge: accelerated pace of discovery transforms marine virology. Nat. Rev. Microbiol..

[4] Páez-Espino D (2016). Uncovering Earth's virome. Nature.

[5] Shi M (2016). Redefining the invertebrate RNA virosphere. Nature.

[6] Dayaram A (2016). Diverse circular replication-associated protein encoding viruses circulating in invertebrates within a lake ecosystem. Infect. Genet. Evol..

[7] Roux S (2016). Ecogenomics and potential biogeochemical impacts of globally abundant ocean viruses. Nature.

[8] Arkhipova K (2018). Temporal dynamics of uncultured viruses: a new dimension in viral diversity. ISME J..

[9] Wilson WH (2017). Genomic exploration of individual giant ocean viruses. ISME J..

[10] Brister JR, Ako-Adjei D, Bao Y, Blinkova O (2015). NCBI viral genomes resource. Nucleic Acids Res..

[11] Páez-Espino D (2017). IMG/VR: a database of cultured and uncultured DNA viruses and retroviruses. Nucleic Acids Res..

[12] Bowers RM (2017). Minimum information about a single amplified genome (MISAG) and a metagenome-assembled genome (MIMAG) of bacteria and archaea. Nat. Biotechnol..

[13] Field D (2008). The minimum information about a genome sequence (MIGS) specification. Nat. Biotechnol..

[14] Yilmaz P (2011). Minimum information about a marker gene sequence (MIMARKS) and minimum information about any (x) sequence (MIxS) specifications. Nat. Biotechnol..

[15] Simmonds P (2017). Consensus statement: virus taxonomy in the age of metagenomics. Nat. Rev. Microbiol..

[16] Ladner JT (2014). Standards for sequencing viral genomes in the era of high-throughput sequencing. MBio.

[17] Thurber RV, Haynes M, Breitbart M, Wegley L, Rohwer F (2009). Laboratory procedures to generate viral metagenomes. Nat. Protoc..

[18] Mokili JL, Rohwer F, Dutilh BE (2012). Metagenomics and future perspectives in virus discovery. Curr. Opin. Virol..

[19] Duhaime MB, Deng L, Poulos BT, Sullivan MB (2012). Towards quantitative metagenomics of wild viruses and other ultra-low concentration DNA samples: a rigorous assessment and optimization of the linker amplification method. Environ. Microbiol..

[20] Wylie TN, Wylie KM, Herter BN, Storch GA (2015). Enhanced virome sequencing using targeted sequence capture. Genome Res..

[21] Allen LZ (2011). Single virus genomics: a new tool for virus discovery. PLoS One.

[22] Martinez-Hernandez F (2017). Single-virus genomics reveals hidden cosmopolitan and abundant viruses. Nat. Commun..

[23] Stepanauskas R (2017). Improved genome recovery and integrated cell-size analyses of individual uncultured microbial cells and viral particles. Nat. Commun..

[24] Houldcroft CJ, Beale MA, Breuer J (2017). Clinical and biological insights from viral genome sequencing. Nat. Rev. Microbiol..

[25] Hingamp P (2013). Exploring nucleo-cytoplasmic large DNA viruses in Tara Oceans microbial metagenomes. ISME J..

[26] Roux S, Hallam SJ, Woyke T, Sullivan MB (2015). Viral dark matter and virus-host interactions resolved from publicly available microbial genomes. Elife.

[27] Kang, H.S. et al. Prophage genomics reveals patterns in phage genome organization and replication. Preprint at *bioRxiv* https://www.biorxiv.org/content/early/2017/03/07/114819 (2017).

[28] Casjens S (2003). Prophages and bacterial genomics: what have we learned so far?. Mol. Microbiol..

[29] López-Pérez M, Haro-Moreno JM, Gonzalez-Serrano R, Parras-Moltó M, Rodriguez-Valera F (2017). Genome diversity of marine phages recovered from Mediterranean metagenomes: size matters. PLoS Genet..

[30] Roux S, Krupovic M, Debroas D, Forterre P, Enault F (2013). Assessment of viral community functional potential from viral metagenomes may be hampered by contamination with cellular sequences. Open Biol..

[31] Luef B (2015). Diverse uncultivated ultra-small bacterial cells in groundwater. Nat. Commun..

[32] Frost LS, Leplae R, Summers AO, Toussaint A (2005). Mobile genetic elements: the agents of open source evolution. Nat. Rev. Microbiol..

[33] Lang AS, Beatty JT (2007). Importance of widespread gene transfer agent genes in alpha-proteobacteria. Trends Microbiol..

[34] Biller SJ (2017). Membrane vesicles in sea water: heterogeneous DNA content and implications for viral abundance estimates. ISME J..

[35] Arndt D (2016). PHASTER: a better, faster version of the PHAST phage search tool. Nucleic Acids Res..

[36] Roux S, Enault F, Hurwitz BL, Sullivan MB (2015). VirSorter: mining viral signal from microbial genomic data. PeerJ.

[37] Amgarten D, Braga LPP, da Silva AM, Setubal JC (2018). MARVEL, a tool for prediction of bacteriophage sequences in metagenomic bins. Front. Genet..

[38] Ren J, Ahlgren NA, Lu YY, Fuhrman JA, Sun F (2017). VirFinder: a novel k-mer based tool for identifying viral sequences from assembled metagenomic data. Microbiome.

[39] Zhao G (2017). VirusSeeker, a computational pipeline for virus discovery and virome composition analysis. Virology.

[40] Páez-Espino D, Pavlopoulos GA, Ivanova NN, Kyrpides NC (2017). Nontargeted virus sequence discovery pipeline and virus clustering for metagenomic data. Nat. Protoc..

[41] Moniruzzaman M (2016). Diversity and dynamics of algal Megaviridae members during a harmful brown tide caused by the pelagophyte, *Aureococcus anophagefferens*. FEMS Microbiol. Ecol..

[42] Sakowski EG (2014). Ribonucleotide reductases reveal novel viral diversity and predict biological and ecological features of unknown marine viruses. Proc. Natl. Acad. Sci. USA.

[43] Marine RL, Nasko DJ, Wray J, Polson SW, Wommack KE (2017). Novel chaperonins are prevalent in the virioplankton and demonstrate links to viral biology and ecology. ISME J..

[44] Schmidt HF, Sakowski EG, Williamson SJ, Polson SW, Wommack KE (2014). Shotgun metagenomics indicates novel family A DNA polymerases predominate within marine virioplankton. ISME J..

[45] Culley AI, Lang AS, Suttle CA (2006). Metagenomic analysis of coastal RNA virus communities. Science.

[46] Needham DM, Sachdeva R, Fuhrman JA (2017). Ecological dynamics and co-occurrence among marine phytoplankton, bacteria and myoviruses shows microdiversity matters. ISME J..

[47] Roux S, Emerson JB, Eloe-Fadrosh EA, Sullivan MB (2017). Benchmarking viromics: an *in silico* evaluation of metagenome-enabled estimates of viral community composition and diversity. PeerJ.

[48] Lorenzi HA (2011). The viral metagenome annotation pipeline (VMGAP): an automated tool for the functional annotation of viral metagenomic shotgun sequencing data. Stand. Genomic Sci..

[49] McNair K (2018). Phage genome annotation using the RAST pipeline. Methods Mol. Biol..

[50] Brister JR (2010). Towards viral genome annotation standards, report from the 2010 NCBI Annotation Workshop. Viruses.

[51] Eddy SR (2011). Accelerated profile HMM searches. PLoS Comput. Biol..

[52] Altschul SF (1997). Gapped BLAST and PSI-BLAST: a new generation of protein database search programs. Nucleic Acids Res..

[53] Söding J (2005). Protein homology detection by HMM-HMM comparison. Bioinformatics.

[54] Reyes AP, Alves JM, Durham AM, Gruber A (2017). Use of profile hidden Markov models in viral discovery: current insights. Adv. Genomics Genet..

[55] Gregory AC (2016). Genomic differentiation among wild cyanophages despite widespread horizontal gene transfer. BMC Genomics.

[56] Duhaime MB (2017). Comparative omics and trait analyses of marine *Pseudoalteromonas* phages advance the phage OTU concept. Front. Microbiol..

[57] Mavrich TN, Hatfull GF (2017). Bacteriophage evolution differs by host, lifestyle and genome. Nat. Microbiol..

[58] Aiewsakun P, Simmonds P (2018). The genomic underpinnings of eukaryotic virus taxonomy: creating a sequence-based framework for family-level virus classification. Microbiome.

[59] Bolduc B (2017). vConTACT: an iVirus tool to classify double-stranded DNA viruses that infect *Archaea* and *Bacteria*. PeerJ.

[60] Mizuno CM, Rodriguez-Valera F, Kimes NE, Ghai R (2013). Expanding the marine virosphere using metagenomics. PLoS Genet..

[61] Bào Y (2017). Implementation of objective PASC-derived taxon demarcation criteria for official classification of filoviruses. Viruses.

[62] Varsani A, Krupovic M (2017). Sequence-based taxonomic framework for the classification of uncultured single-stranded DNA viruses of the family Genomoviridae. Virus Evol..

[63] Rohwer F, Edwards R (2002). The phage proteomic tree: a genome-based taxonomy for phage. J. Bacteriol..

[64] Lavigne R (2009). Classification of Myoviridae bacteriophages using protein sequence similarity. BMC Microbiol..

[65] Nishimura Y (2017). ViPTree: the viral proteomic tree server. Bioinformatics.

[66] Meier-Kolthoff JP, Göker M (2017). VICTOR: genome-based phylogeny and classification of prokaryotic viruses. Bioinformatics.

[67] Baltimore D (1971). Expression of animal virus genomes. Bacteriol. Rev..

[68] Edwards RA, McNair K, Faust K, Raes J, Dutilh BE (2016). Computational approaches to predict bacteriophage-host relationships. FEMS Microbiol. Rev..

[69] Mihara T (2016). Linking virus genomes with host taxonomy. Viruses.

[70] Villarroel J (2016). HostPhinder: a phage host prediction tool. Viruses.

[71] Garcia-Heredia I (2012). Reconstructing viral genomes from the environment using fosmid clones: the case of haloviruses. PLoS One.

[72] Roux S (2014). Ecology and evolution of viruses infecting uncultivated SUP05 bacteria as revealed by single-cell- and meta-genomics. Elife.

[73] Labonté JM (2015). Single-cell genomics-based analysis of virus-host interactions in marine surface bacterioplankton. ISME J..

[74] Galiez C, Siebert M, Enault F, Vincent J, Söding J (2017). WIsH: who is the host? Predicting prokaryotic hosts from metagenomic phage contigs. Bioinformatics.

[75] Ahlgren NA, Ren J, Lu YY, Fuhrman JA, Sun F (2017). Alignment-free *d*_2_* oligonucleotide frequency dissimilarity measure improves prediction of hosts from metagenomically-derived viral sequences. Nucleic Acids Res..

[76] Reyes A, Wu M, McNulty NP, Rohwer FL, Gordon JI (2013). Gnotobiotic mouse model of phage-bacterial host dynamics in the human gut. Proc. Natl. Acad. Sci. USA.

[77] Lima-Mendez G (2015). Determinants of community structure in the global plankton interactome. Science.

[78] Dutilh BE (2014). A highly abundant bacteriophage discovered in the unknown sequences of human faecal metagenomes. Nat. Commun..

[79] Coenen AR, Weitz JS (2018). Limitations of correlation-based inference in complex virus-microbe communities. mSystems.

[80] Gao NL (2018). MVP: a microbe-phage interaction database. Nucleic Acids Res..

[81] Aziz RK, Dwivedi B, Akhter S, Breitbart M, Edwards RA (2015). Multidimensional metrics for estimating phage abundance, distribution, gene density, and sequence coverage in metagenomes. Front. Microbiol..

[82] Adams MJ (2017). 50 years of the International Committee on Taxonomy of Viruses: progress and prospects. Arch. Virol..

[83] Reyes A (2015). Gut DNA viromes of Malawian twins discordant for severe acute malnutrition. Proc. Natl. Acad. Sci. USA.

[84] Shendure J (2005). Accurate multiplex polony sequencing of an evolved bacterial genome. Science.

[85] Margulies M (2005). Genome sequencing in microfabricated high-density picolitre reactors. Nature.

[86] Angly FE (2006). The marine viromes of four oceanic regions. PLoS Biol..

[87] Lima-Mendez G, Van Helden J, Toussaint A, Leplae R (2008). Prophinder: a computational tool for prophage prediction in prokaryotic genomes. Bioinformatics.

[88] Reyes A (2010). Viruses in the faecal microbiota of monozygotic twins and their mothers. Nature.

[89] Yoon HS (2011). Single-cell genomics reveals organismal interactions in uncultivated marine protists. Science.

[90] Andrewes CH (1955). The classification of viruses. J. Gen. Microbiol..

[91] Lwoff A, Horne R, Tournier P (1962). A system of viruses. Cold Spring Harb. Symp. Quant. Biol..

[92] Lwoff A (1964). The new provisional committee on nomenclature of viruses. Int. Bull. Bacteriol. Nomencl. Taxon..

[93] King AMQ (2018). Changes to taxonomy and the International Code of Virus Classification and Nomenclature ratified by the International Committee on Taxonomy of Viruses. Arch. Virol..

