# Supplementary figures and images for: Minimum Information about an Uncultivated Virus Genome (MIUViG)
Source: Nat Biotechnol. 2018 Dec 17;37(1):29–37. doi: 10.1038/nbt.4306 (PMC6871006; doi:10.1038/nbt.4306)

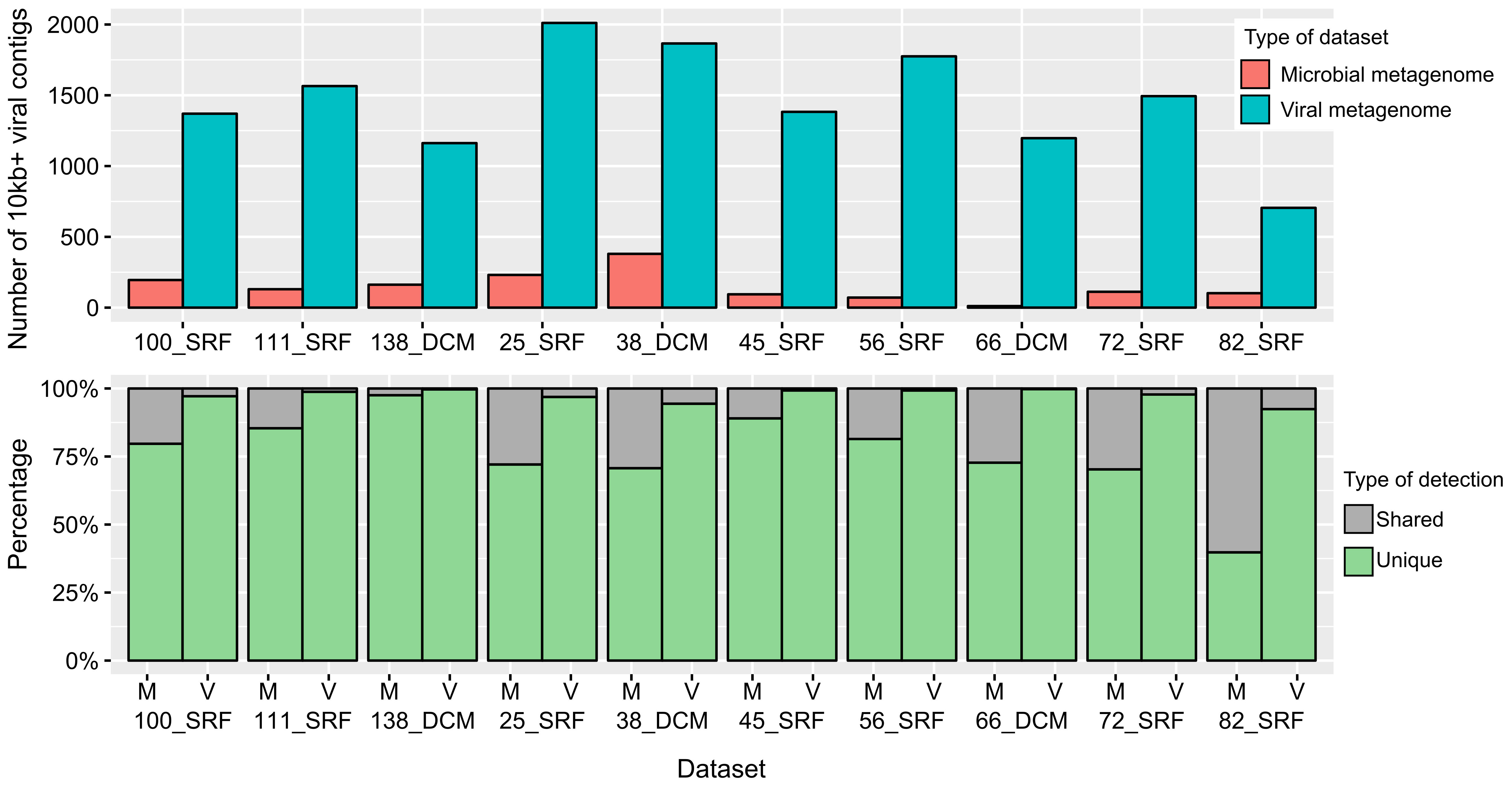

Supplement: Comparison of UViG recovery from microbial (“M”) and viral (“V”) metagenomes originating from the same Tara Oceans samples. — Top panel represents the number of distinct virus contigs ≥ 10kb identified in each dataset. The bottom panel depicts the ratio of “shared”, i.e., detected in both viral and microbial fraction of the sample, and “unique”, i.e., detected only in one fraction, contigs in each microbial and viral fraction. Datasets were originally analyzed in refs. 1,2. SRF: surface, DCM: deep chlorophyll maximum. [file 41587_2019_Article_BFnbt4306_Fig4_ESM.jpg]

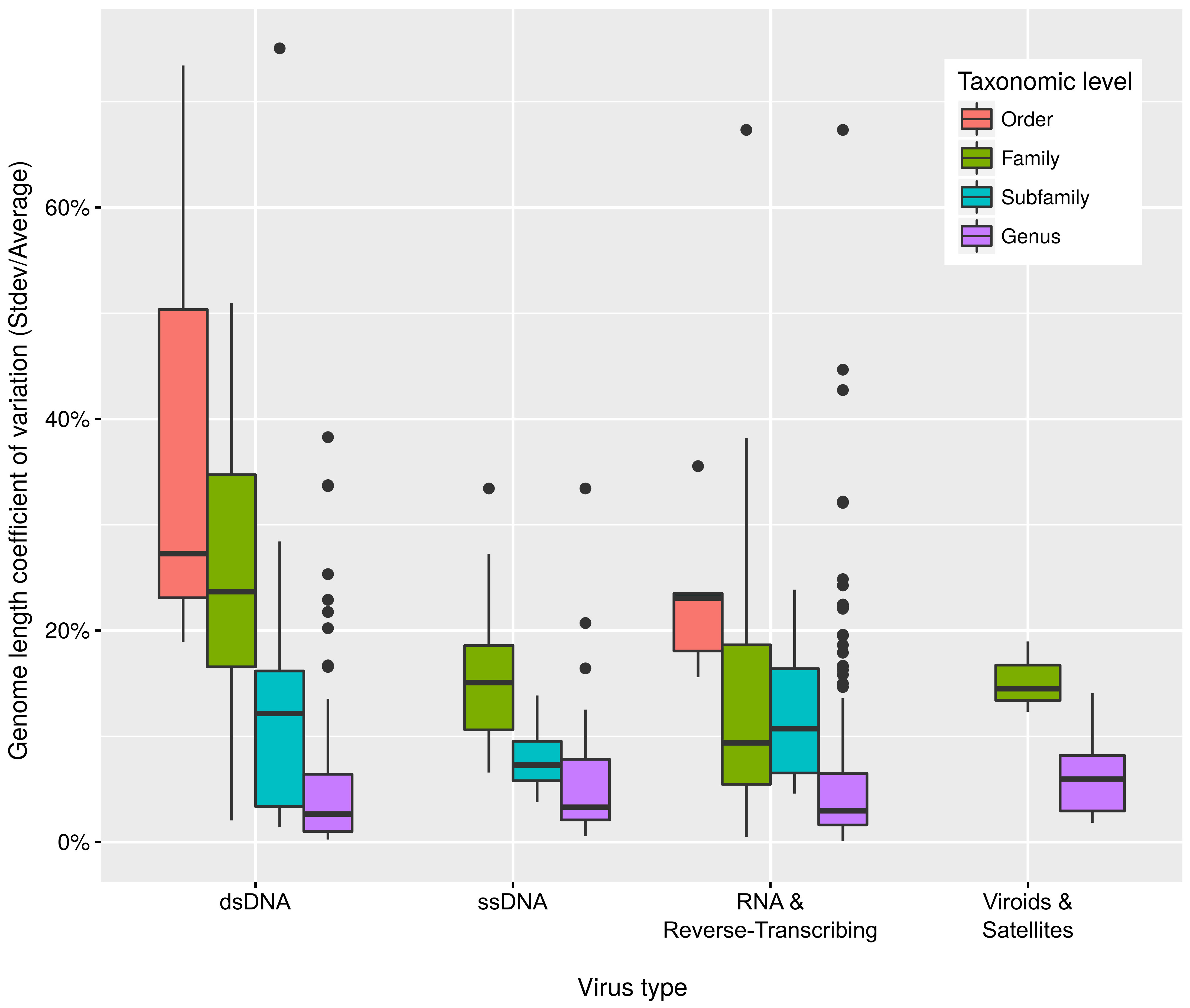

Supplement: Genome size variation for different types of viruses and different taxonomic levels. — Genome length of virus genomes from NCBI RefSeq were compared at different taxonomic ranks and are presented separately for four main types of viruses (dsDNA, ssDNA, RNA and reverse-transcribing RNA, viroids and satellites). Genome length variation was calculated as a coefficient of variation, i.e. standard deviation of genome length in the group divided by average genome length in the grouop (for groups with >1 genome). Underlying data are available in Supplementary Table 5. Boxplots lower and upper hinges correspond to the first and third quartiles (the 25th and 75th percentiles), while whisker extend from the nearest hinge to the smallest/largest value no further than 1.5 * IQR from the hinge (where IQR is the inter-quartile range, or distance between the first and third quartiles). dsDNA: double-stranded DNA; ssDNA: single-stranded DNA. [file 41587_2019_Article_BFnbt4306_Fig5_ESM.jpg]

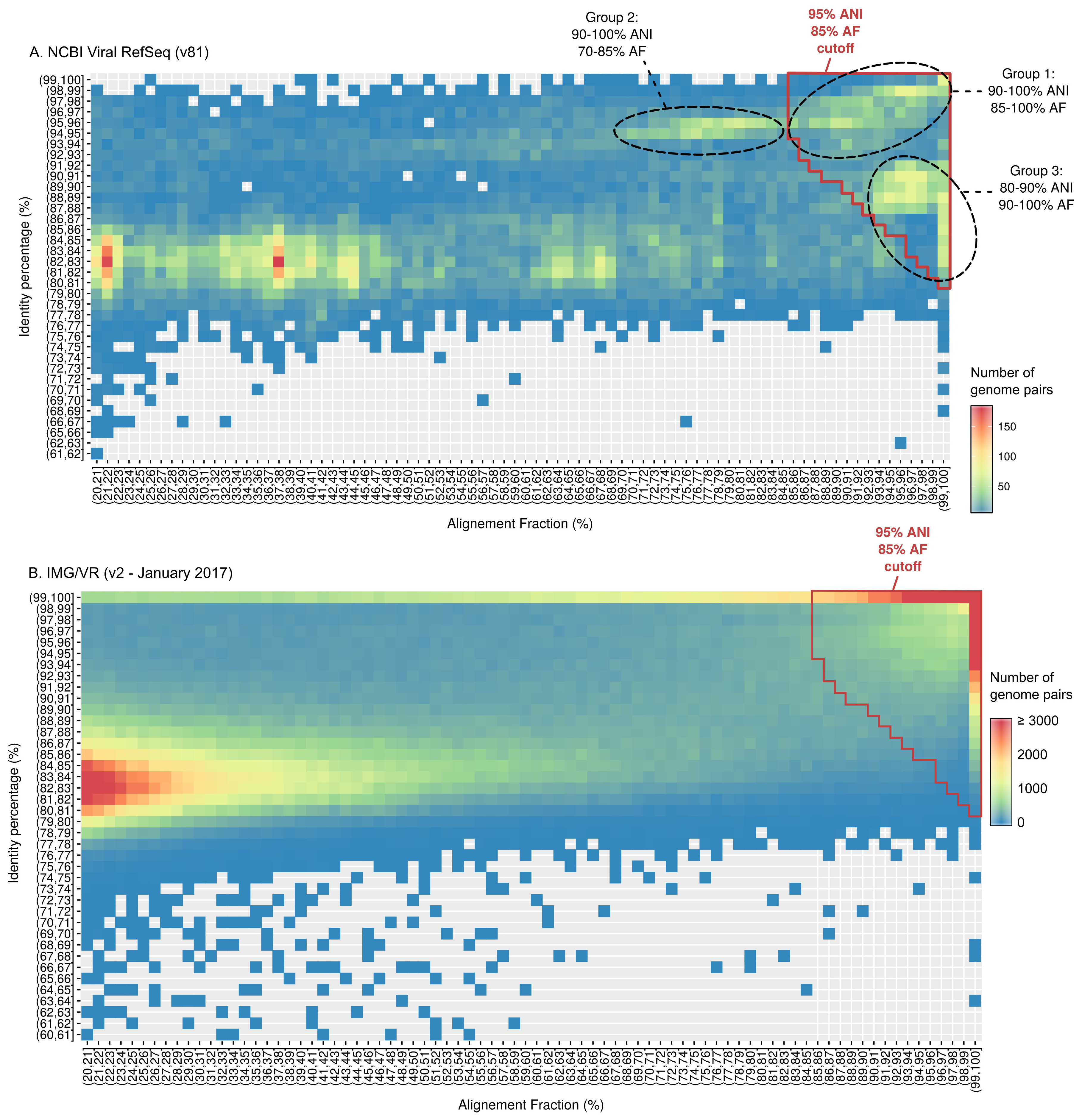

Supplement: Pairwise average nucleotide identity (ANI) and alignment fraction (AF) for NCBI Viral RefSeq genomes and IMG/VR. — Only genome pairs with ANI >60% and AF >20% were considered. ANI and AF were binned in 1% intervals, and are represented here as a heatmap (i.e. cell coloring represents the number of pairwise comparisons at the corresponding ANI and AF intervals). On the top right corner (i.e., AF and ANI close to 100%), three main groups of genome pairs are delineated with black dashed circles, and the proposed standard cutoff is highlighted in dark red. Note that for this clustering, the cutoff was applied as follows: pairs of genomes with ≥ 85% AF were first selected, and whole genome (wg) ANI was then calculated by multiplying the observed ANI by the observed AF. This wgANI was then compared to the corresponding whole genome ANI cutoff (i.e. 95% ANI * 85% AF = 80.75% wgANI). This allows for hits with ≤ 95% ANI but ≥ 85 % AF to be considered as well, i.e. a pair of genomes with 90% ANI on 100% AF would be considered as “passing” the cutoff. Examples of genome comparisons for each group are presented in Supplementary Fig. 4. [file 41587_2019_Article_BFnbt4306_Fig6_ESM.jpg]

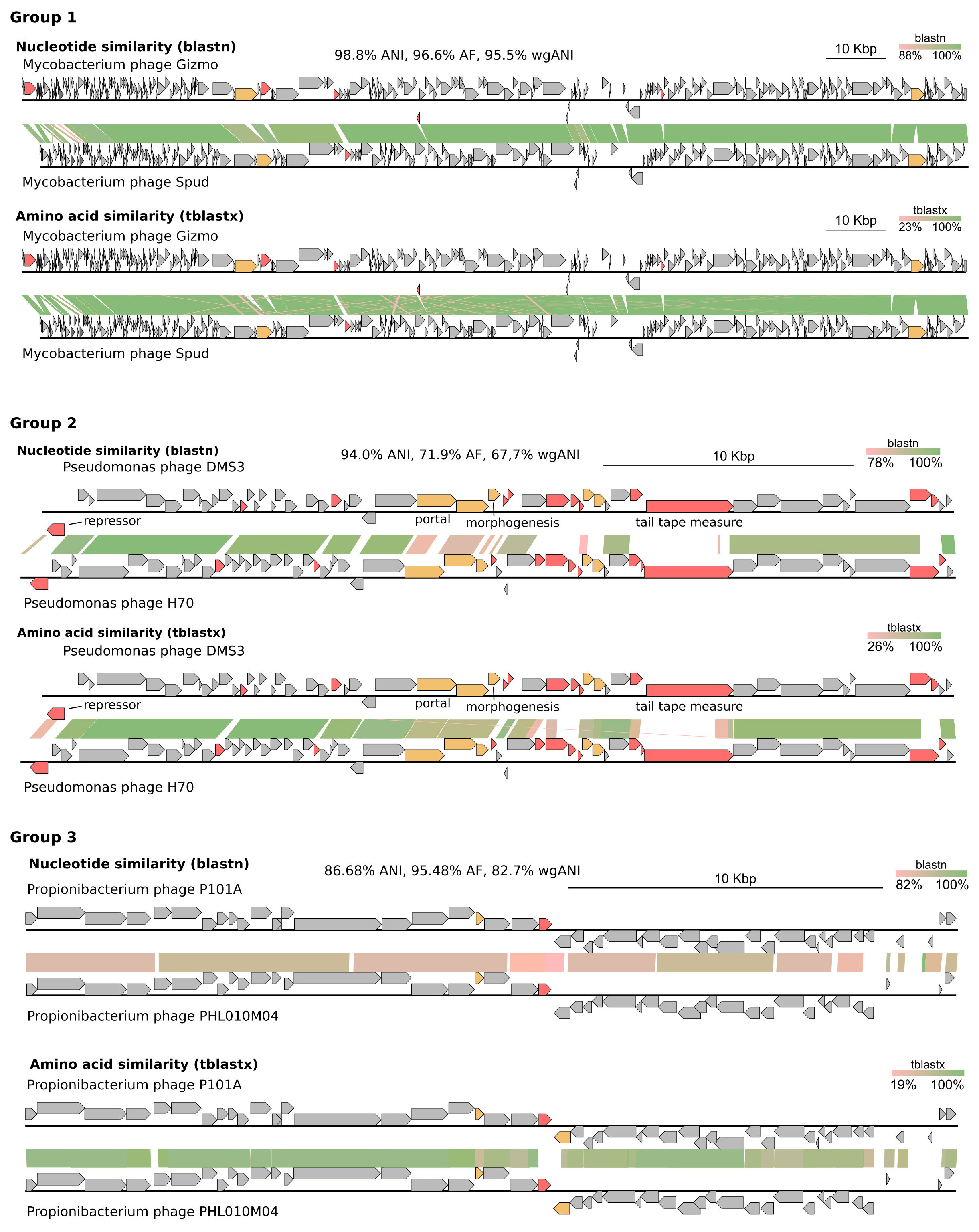

Supplement: Examples of pairwise genome comparisons from the three groups of genome pairs highlighted in Supplementary Figure 3. — For each example, nucleotide similarity (blastn) and amino acid similarity (tblastx) are displayed, alongside the ANI, AF, and wgANI (i.e. ANI over the whole length of the shorter genome). AF, alignment fraction; ANI, average nucleotide identity; wgANI, whole-genome average nucleotide identity. [file 41587_2019_Article_BFnbt4306_Fig7_ESM.jpg]

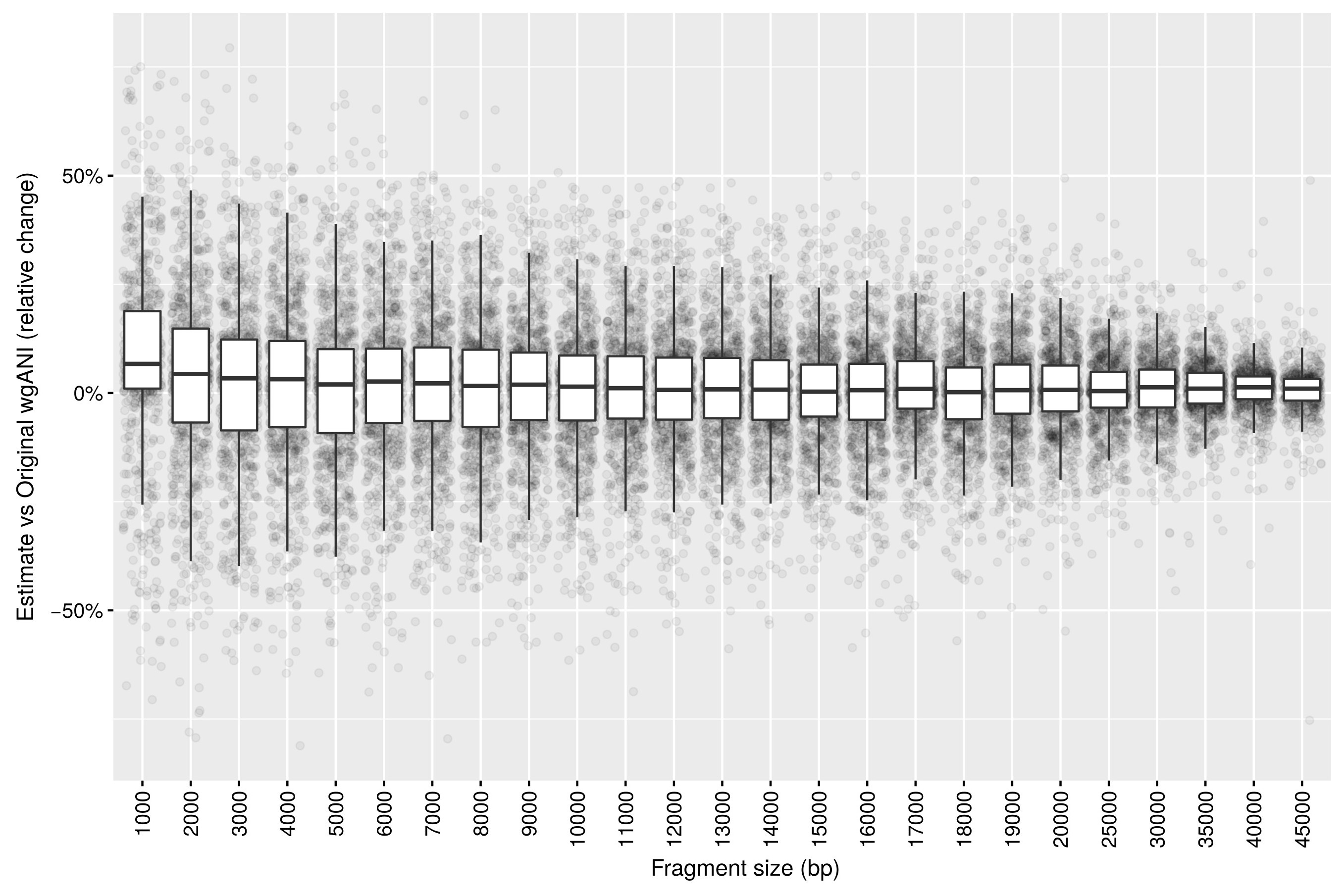

Supplement: Estimation of whole genome ANI from fragmented genomes. — To evaluate the impact of genome fragmentation on whole-genome average nucleotide identity (wgANI) estimation, pairs of genomes from NCBI RefSeq with wgANI ≥ 70% and ≥ 20kb were selected, random fragments were generated (from 1 to 45kb) from one of the two genomes, and then compared to the other complete genome. The resulting estimated wgANI between the fragment and complete genome was then compared with the original values estimated from the two complete genomes (y-axis). Boxplots lower and upper hinges correspond to the first and third quartiles (the 25th and 75th percentiles), while whisker extend from the nearest hinge to the smallest/largest value no further than 1.5 * IQR from the hinge (where IQR is the inter-quartile range, or distance between the first and third quartiles). [file 41587_2019_Article_BFnbt4306_Fig8_ESM.jpg]
