# Supplementary material for: Minimum Information about an Uncultivated Virus Genome (MIUViG)
Source: Nat Biotechnol. 2018 Dec 17;37(1):29–37. doi: 10.1038/nbt.4306 (PMC6871006; doi:10.1038/nbt.4306)
Supplement: Supplementary Notes — 1–4 (PDF 196 kb) [file 41587_2019_BFnbt4306_MOESM35_ESM.pdf]

Supplementary Note 1. Comparison of UViG recovery from viral and microbial size fraction metagenomes.

The broad range of datasets from which UViGs can be extracted (Fig. 2) reflects both the pervasiveness of viruses and their critical importance in multiple fields, such as evolutionary biology, microbial ecology, and infectious diseases. Some of these techniques are better suited towards addressing specific biological questions but from the virus discovery standpoint, these approaches are mostly complementary. To highlight the differences and complementarity between approaches, we compared the number of large UViGs (here virus contigs ≥ 10kb) assembled from virus-targeted and microbial cell-targeted metagenomes from the same samples obtained through the *Tara* Oceans expedition[1,2], after we subsampled them to the same number of reads (Supplementary Fig. 1). Metagenomes targeting the nominal virus fraction yielded, on average, 20 times more UViGs than their microbe-targeted counterparts. However, at the current sequencing depth, UViGs derived from microbial metagenomes were not subsets of the UViGs identified in the viral metagenomes, with an average 74% of the UViGs unique to the microbial fraction (range: 34–98%). This comparison illustrates how integrating virus sequences from samples across different size fractions and/or processed with different techniques is highly valuable for exploring the virus genome sequence space[3].

Nature Biotechnology: doi:10.1038/nbt.4306

Supplementary Note 2. UViG quality category assignment for the Global Ocean Virome dataset

Sequences from the Global Ocean Virome dataset were analyzed to illustrate how UViGs can be assigned to the different quality categories. Of note, the methods and approaches used here are meant as an example but not as a step-by-step guide to be strictly followed, especially since most of these approaches are continuously improving and new tools are frequently developed. The Global Ocean Virome dataset already included 15,222 non-redundant "populations" (i.e. vOTUs) defined from 24,353 virus contigs, which had been annotated, classified, and for which hosts were predicted[4]. These data were used to assign quality tiers, i.e. genome fragments, high-quality draft genomes, and finished genomes, to the representative UViG of each vOTU.

All these UViGs were initially selected because their total length was ≥ 10kb, however they can represent varying degrees of completeness (if estimated), and it is thus important to assess and report completeness as much as possible so that follow-up analyses can use their own threshold (Supplementary Table 6). Two different analyses can be conducted to identify high-quality draft genomes: (i) identification of putative complete genomes assembled as circular contigs or linear contigs with inverted terminal repeats, and (ii) affiliation of these UViGs to family or genus-rank groups from which expected genome size can be derived. For the former, the circular contigs representing putative complete genomes were already identified in the dataset, and the same list was used here[4]. For the latter, UViGs were previously classified in genus-rank clusters (viral clusters or VC) that were used to derive an expected genome size. The average length of all complete and predicted complete genomes from a genus-rank cluster was taken when the relative standard deviation of these genome lengths was ≤ 15%[4]. For clusters for which genome lengths displayed a standard deviation > 15%, we considered that no reliable expected genome size could be derived. We also used marker genes from the Viral Orthologous Groups database (http://vogdb.org/, v83, HMMER3 with score ≥ 50) to complete these UViGs affiliations. UViGs were considered as affiliated when at least 2 marker genes affiliations were consistent. An expected genome size was derived for all marker-gene affiliations available at the genus rank. UViGs which total length was estimated to represent ≥ 90% of the expected genome size were classified as high-quality drafts, in addition to the ones predicted as complete. Overall, 517 UViGs were high-quality draft genomes, and 14,705 were genome fragments (Supplementary Table 6).

Finally, three high-quality draft UViGs were selected for manual review and annotation, and transition to "finished genome" status. The selected UViGs were ones represented by a single contig predicted to represent a complete genome, affiliated to some of the most abundant VCs lacking an isolate reference, and when possible detected across multiple samples and with a host prediction. For these UViGs, namely GOV_bin_630 (VC_3), GOV_bin_2604 (VC_8), and GOV_bin_556 (VC_17), the contigs were manually reviewed and annotated to (i) ensure that these contigs did not include multiple copies of single copy of marker genes, (ii) improve affiliation through a comparison of

predicted proteins to updated databases, namely PDB[5] CDD[6], PFAM[7], and SCOpe[8], using the HHPred web server[9], and (iii) classify the affiliated genes in relevant functional categories based on ViralZone DB[10]. Eventually, the final dataset was composed of 3 finished genomes, 514 high-quality draft genomes, and 14,705 genome fragments.

Supplementary Note 3. Evaluation of potential "universal cutoffs" for viral operational taxonomic units (vOTUs)

Because species-rank groupings represent the primary data for many downstream ecological, evolutionary, and functional studies, viral operational taxonomic units (vOTUs), have been routinely defined and used in the literature. To date, most published studies used a combination of minimum average nucleotide identity (ANI) and minimum percentage of shared genes or minimum alignment fraction (AF), with cutoffs varying from 90 to 95% ANI, and from 20% to 100% AF (e.g. [1,11–13]). Criteria for defining species can inform cutoffs for defining uncultivated viral genomes (UViGs) vOTU, however defining cutoffs requires a first classification of these UViGs in a group for which species criteria are available (Supplementary Table 3). To process datasets for which no such classification is available, "universal" cutoffs should be established. We recognize that the methods and thresholds used for defining vOTUs will be highly debated, similar to debates on microbial OTUs (e.g. [14]), and no universal standard can be proclaimed a priori, yet comparative analysis of currently available isolate virus genomes can suggest possible cutoffs.

Pairwise comparisons of nucleotide sequences were computed for all virus genomes from NCBI RefSeq (v81, n=9,158), and ANI and AF (relative to the shorter genome) were calculated using MUMMER[15]. We then looked at the distribution of ANI and AF specifically across genome pairs involving closely related genomes (ANI ≥ 60%, Supplementary Figure 3). From these, three groups of genome pairs can be distinguished. In the first group (Group 1 in Supplementary Figs. 3–4), genomes are (nearly) identical over (nearly) the complete genome length (90–100% ANI, 85–100% AF). In the second group (Group 2 in Supplementary Figs. 3–4), genomes have comparably high identity (90-100% ANI), but with a smaller alignment fraction (70–85% AF), highlighting differences in gene content and thus putative phenomic features. This type of evolutionary mode involving a high gene flux and more unique genes than would be expected based on the sequence similarity of shared genes was recently described as primarily associated with temperate phages[16]. Finally, in the third group (Group 3 in Supplementary Figs. 3–4), genomes display a lower level of similarity (80–90% ANI) over (nearly) the complete genome length (90–100% AF), suggesting that although these viruses are more divergent than the ones in Group 1, they still harbor a similar gene repertoire.

To obtain vOTUs gathering distinct lineages with more genetic exchange within members of the group than with other groups, cutoffs of ≥ 95% ANI over ≥ 85% AF could be applied. These demarcations would keep together genomes from Group 1 and Group 3, while keeping separated the ones from Group 2 (high gene flux, Supplementary Fig. 3A). Notably, based on the sequences available in the IMG/VR database (v2, January 2017), which consists of a much larger dataset (> 250,000 sequences) but covers mostly dsDNA and few if any ssDNA or RNA viruses, the vast majority of the genome pairs would be in the Group 1 (high ANI and high AF, see Supplementary Figure 3B). The proposed cutoffs would thus lead to vOTUs consistent with the published literature.

Nature Biotechnology: doi:10.1038/nbt.4306

Supplementary Note 4. Distribution and abundance of UViGs.

Abundance estimates of a vOTU across datasets provide valuable information on the distribution and potential ecological niche of the virus. The relative abundance and distribution of a virus can be estimated through short-read metagenome mapping. However, thresholds must be applied to (i) the nucleotide identity between the read and UViG sequence, and (ii) the percentage of the representative UViG sequence covered by metagenome reads. Both parameters are critical to avoid false-positive detection[17–19]. Alternatively, pseudo-alignment and abundance estimation through expectation-maximization as implemented e.g. in FastViromeExplorer[20] can be used instead of coverage estimation through read mapping, with similar cutoffs applied on the coverage along the genome and total number of mapped reads.

The specific thresholds for nucleotide identity and coverage of the reference genome can be adjusted depending on the scientific objectives of a given study. For instance, increasing the coverage threshold from 10% to 75% led to a lower rate of incorrect detection (false discovery rate decreased from 8% to 0%) but at the cost of a lower sensitivity (decreased from 88% to 82%, based on simulated datasets from ref. [18]). Thus, when reporting read mapping-based distributions and/or relative abundances, it is important to report the nucleotide identity and coverage thresholds, and provide an estimate of false-positive and false-negative rates for the combined thresholds, either computed *de novo* or extracted from the literature, e.g. from refs [18,19]. Finally, two important caveats should be considered when using read mapping to estimate virus distribution and relative abundance: (i) some amplification methods produce non-quantitative datasets, in which coverage can not be interpreted as relative abundance (Box #1), and (ii) there are currently no guidelines for integrating coverage data from different size fractions.

Nature Biotechnology: doi:10.1038/nbt.4306

Supplementary Figures (legends)

Supplementary Figure 1. Comparison of UViG recovery from microbial (“M”) and viral (“V”) metagenomes originating from the same Tara Oceans samples. Top panel represents the number of distinct virus contigs ≥ 10kb identified in each dataset, and the bottom panel depicts the ratio of “shared” (i.e. detected in both viral and microbial fraction of the sample) and “unique” (detected only in one fraction) contigs in each fraction. Datasets were originally analyzed in ref. [1,2]. SRF: surface, DCM: deep chlorophyll maximum.

Supplementary Figure 2. Genome length variation for different types of viruses and different taxonomic ranks. Genome length of virus genomes from NCBI RefSeq were compared at different taxonomic ranks and are presented separately for four main types of viruses (dsDNA, ssDNA, RNA and reverse-transcribing, viroids and satellites). Genome length variation was calculated as a coefficient of variation at the genus rank, i.e. standard deviation of genome length in the genus divided by average genome length in the genus (for genera with > 1 genomes). Underlying data are available in Supplementary Table 5. Boxplots lower and upper hinges correspond to the first and third quartiles (the 25th and 75th percentiles), while whisker extend from the nearest hinge to the smallest/largest value no further than 1.5 * IQR from the hinge (where IQR is the inter-quartile range, or distance between the first and third quartiles). dsDNA: double-stranded DNA; ssDNA: single-stranded DNA.

Supplementary Figure 3. Pairwise Average Nucleotide Identity (ANI) and Alignment Fraction (AF) for NCBI Viral RefSeq genomes (A) and IMG/VR (B). Only genome pairs with ANI >60% and AF >20% were considered. ANI and AF were binned in 1% intervals, and are represented here as a heatmap (i.e. cell coloring represents the number of pairwise comparisons at the corresponding ANI and AF intervals). On the top right corner (i.e. AF and ANI close to 100%), three main groups of genome pairs are delineated with black dashed circles, and the proposed standard cutoff is highlighted in dark red. Note that for this clustering, the cutoff was applied as follows: pairs of genomes with ≥ 85% AF were first selected, and whole genome (wg) ANI was then calculated by multiplying the observed ANI by the observed AF. This wgANI was then compared to the corresponding whole genome ANI cutoff (i.e. 95% ANI * 85% AF = 80.75% wgANI). This allows for hits with ≤ 95% ANI but ≥ 85 % AF to be considered as well, i.e. a pair of genomes with 90% ANI on 100% AF would be considered as “passing” the cutoff. Examples of genome comparisons for each group are presented in Supplementary Figure 4 (from NCBI Viral RefSeq).

Supplementary Figure 4. Examples of pairwise genome comparisons from the three groups of genome pairs highlighted on Supplementary Figure 3. For each example, nucleotide similarity

Nature Biotechnology: doi:10.1038/nbt.4306

(blastn) and amino acid similarity (tblastx) are displayed, alongside the ANI, AF, and wgANI (i.e. ANI over the whole length of the shorter genome).

Supplementary Figure 5. Estimation of whole genome (wg) ANI from fragmented genomes. To evaluate the impact of genome fragmentation on wgANI estimation, pairs of genomes from NCBI RefSeq with wgANI ≥ 70% and ≥ 20kb were selected, random fragments were generated (from 1 to 45kb) from one of the two genomes, and then compared to the other complete genome. The resulting wgANI between the fragment and complete genome was then compared with the original values estimated from the two complete genomes (y-axis). Boxplots lower and upper hinges correspond to the first and third quartiles (the 25th and 75th percentiles), while whisker extend from the nearest hinge to the smallest/largest value no further than 1.5 * IQR from the hinge (where IQR is the inter-quartile range, or distance between the first and third quartiles).

Nature Biotechnology: doi:10.1038/nbt.4306

Supplementary Tables (legends)

Supplementary Table 1. List of mandatory and optional metadata for UViGs. Mandatory metadata are highlighted in blue. The status of metadata indicates if identical or similar information is included in the MIMAG / MISAG standards, with virus-specific metadata highlighted in orange, and metadata adapted for UViGs in purple. If one of the mandatory metadata is missing, the value should be set as "Not applicable" for metadata that cannot be evaluated, or "Missing – Not collected" for the ones that could be assessed but for which the result is not currently available. MIMAG: metagenome-assembled genome; MISAG: minimum information about a single amplified genome. ANI: Average Nucleotide Identity. AF: Alignment Fraction.

Supplementary Table 2. List of metadata from previous standards relevant for UViGs[21]. The last 3 columns include information about whether an item is mandatory (M), conditional mandatory (C), optional (X), environment-dependent (E) or not applicable (-) in the MIMAG, MISAG, and MIUViG checklists. Items for which the MIUViG requirement differed from MIMAG and MISAG requirements are highlighted in yellow.

Supplementary Table 3. Comparison between UViGs categories and the quality categories proposed for small DNA/RNA virus whole-genome sequencing for epidemiology and surveillance by Ladner et al. [22].

Supplementary Table 4. List and characteristics of tools used to identify virus sequences in mixed datasets, published or updated since 2012[23–31].

Supplementary Table 5. Variation in genome length for virus families and genera with 2 or more genomes, from NCBI RefSeq v83.

Supplementary Table 6. List of potential marker genes for virus orders, families, or genera, based on the VOGdb v83 (http://vogdb.org/).

Supplementary Table 7. List of UViGs from the GOV dataset[4] considered as high-quality drafts or finished genomes. Example of UViGs classified as genome fragments with varying size and completeness estimations are also included at the bottom of the table. For genome fragments for which no complete genome is available, the expected genome size is displayed as greater than the size of the largest contig in the cluster (e.g. "> 20,000bp"), and no estimated completeness can be provided for these contigs.

Supplementary Table 8. List of databases providing collections of HMM profiles for virus protein families[32–35]. This topic has been recently reviewed in Reyes et al. [36].

Supplementary Table 9. Current species demarcation criteria from ICTV 9th and 10th reports.

Supplementary Table 10. Approaches available for in silico host prediction[18,37–42].

Nature Biotechnology: doi:10.1038/nbt.4306

Supplementary Material References

1. Brum, J. *et al.* Ocean plankton. Patterns and ecological drivers of ocean viral communities. *Science* **348,** 1261498 (2015).

2. Sunagawa, S. *et al.* Ocean plankton. Structure and function of the global ocean microbiome. *Science* **348,** 1261359 (2015).

3. López-Pérez, M., Haro-Moreno, J. M., Gonzalez-Serrano, R., Parras-Moltó, M. & Rodriguez-Valera, F. Genome diversity of marine phages recovered from Mediterranean metagenomes: Size matters. *PLoS Genet.* **13,** e1007018 (2017).

4. Roux, S. *et al.* Ecogenomics and potential biogeochemical impacts of uncultivated globally abundant ocean viruses. *Nature* **537,** 689–93 (2016).

5. Berman, H., Henrick, K. & Nakamura, H. Announcing the worldwide Protein Data Bank. *Nat. Struct. Biol.* **10,** 980 (2003).

6. Marchler-Bauer, A. *et al.* CDD/SPARCLE: Functional classification of proteins via subfamily domain architectures. *Nucleic Acids Res.* **45,** D200–D203 (2017).

7. Finn, R. D. *et al.* The Pfam protein families database: Towards a more sustainable future. *Nucleic Acids Res.* **44,** D279–D285 (2016).

8. Chandonia, J. M., Fox, N. K. & Brenner, S. E. SCOPe: Manual Curation and Artifact Removal in the Structural Classification of Proteins – extended Database. *J. Mol. Biol.* **429,** 348–355 (2017).

9. Zimmermann, L. *et al.* A Completely Reimplemented MPI Bioinformatics Toolkit with a New HHpred Server at its Core. *J. Mol. Biol.* 1–7 (2017). doi:10.1016/j.jmb.2017.12.007

10. Hulo, C. *et al.* ViralZone: a knowledge resource to understand virus diversity. *Nucleic Acids Res.* **39,** D576-82 (2011).

11. Mizuno, C. M., Rodriguez-Valera, F., Kimes, N. E. & Ghai, R. Expanding the marine virosphere using metagenomics. *PLoS Genet.* **9,** e1003987 (2013).

12. Páez-Espino, D. *et al.* Uncovering Earth's virome. *Nature* **536,** 425–430 (2016).

13. Martinez-Hernandez, F. *et al.* Single-virus genomics reveals hidden cosmopolitan and abundant viruses. *Nat. Commun.* **8,** 15892 (2017).

14. Edgar, R. C. Accuracy of microbial community diversity estimated by closed- and open-reference OTUs. *PeerJ* **5,** e3889 (2017).

15. Delcher, A. L., Salzberg, S. L. & Phillippy, A. M. Using MUMmer to identify similar regions in large sequence sets. *Curr. Protoc. Bioinforma.* **00:10.3,** 10.3.1–10.3.18 (2003).

16. Mavrich, T. N. & Hatfull, G. F. Bacteriophage evolution differs by host, lifestyle and genome. *Nat. Microbiol.* **2,** 17112 (2017).

17. Emerson, J. B. *et al.* Dynamic viral populations in hypersaline systems as revealed by metagenomic assembly. *Appl. Environ. Microbiol.* **78,** 6309–20 (2012).

18. Roux, S., Emerson, J. B., Eloe-Fadrosh, E. A. & Sullivan, M. B. Benchmarking viromics: An in silico evaluation of metagenome-enabled estimates of viral community composition and diversity. *PeerJ* **5,** e3817 (2017).

19. Aziz, R. K., Dwivedi, B., Akhter, S., Breitbart, M. & Edwards, R. A. Multidimensional metrics for estimating phage abundance, distribution, gene density, and sequence coverage in metagenomes. *Front. Microbiol.* **6,** 381 (2015).

20. Tithi, S. S., Aylward, F. O., Jensen, R. V. & Zhang, L. FastViromeExplorer: a pipeline for virus and phage identification and abundance profiling in metagenomics data. *PeerJ* **6,** e4227 (2018).

21. Bowers, R. M. *et al.* Minimum information about a single amplified genome (MISAG) and a metagenome-assembled genome (MIMAG) of bacteria and archaea. *Nat. Biotechnol.* **35,** 725–731 (2017).

22. Ladner, J. T. *et al.* Standards for sequencing viral genomes in the era of high-throughput sequencing. *MBio* **5,** e01360–e01314 (2014).

23. Akhter, S., Aziz, R. K. & Edwards, R. A. PhiSpy: a novel algorithm for finding prophages in bacterial genomes that combines similarity- and composition-based strategies. *Nucleic Acids Res.* **40,** 1–13 (2012).

24. Arndt, D. *et al.* PHASTER: a better, faster version of the PHAST phage search tool. *Nucleic Acids Res.* **44,** 1–6 (2016).

25. Zhou, Y., Liang, Y., Lynch, K. H., Dennis, J. J. & Wishart, D. S. PHAST: a fast phage search tool. *Nucleic Acids Res.* **39,** W347-52 (2011).

26. Roux, S., Enault, F., Hurwitz, B. L. & Sullivan, M. B. VirSorter: mining viral signal from microbial genomic data. *PeerJ* **3,** e985 (2015).

27. Lima-Mendez, G., Van Helden, J., Toussaint, A. & Leplae, R. Prophinder: a computational tool for prophage prediction in prokaryotic genomes. *Bioinformatics* **24,** 863–5 (2008).

28. Ren, J., Ahlgren, N. A., Lu, Y. Y., Fuhrman, J. A. & Sun, F. VirFinder: a novel k-mer based tool for identifying viral sequences from assembled metagenomic data. *Microbiome* **5,** 1–20 (2017).

29. Zhao, G. *et al.* VirusSeeker, a computational pipeline for virus discovery and virome composition analysis. *Virology* **503,** 21–30 (2017).

30. Naccache, S. N. *et al.* A cloud-compatible bioinformatics pipeline for ultrarapid pathogen identification from next-generation sequencing of clinical samples. *Genome Res.* **24,** 1180–1192 (2014).

31. Páez-Espino, D., Pavlopoulos, G. A., Ivanova, N. N. & Kyrpides, N. C. Nontargeted virus sequence discovery pipeline and virus clustering for metagenomic data. *Nat. Protoc.* **12,** 1673–1682 (2017).

32. Grazziotin, A. L., Koonin, E. V. & Kristensen, D. M. Prokaryotic Virus Orthologous Groups (pVOGs): A resource for comparative genomics and protein family annotation. *Nucleic Acids Res.* **45,** D491–D498 (2017).

33. Huerta-Cepas, J. *et al.* EGGNOG 4.5: A hierarchical orthology framework with improved functional annotations for eukaryotic, prokaryotic and viral sequences. *Nucleic Acids Res.* **44,** D286–D293 (2016).

34. Skewes-Cox, P., Sharpton, T. J., Pollard, K. S. & DeRisi, J. L. Profile hidden Markov models for the detection of viruses within metagenomic sequence data. *PLoS One* **9,** (2014).

35. Llorens, C. *et al.* The Gypsy Database (GyDB) of Mobile Genetic Elements: Release 2.0. *Nucleic Acids Res.* **39,** 70–74 (2011).

36. Reyes, A., P. Alves, J. M., Durham, A. M. & Gruber, A. Use of profile hidden Markov models in viral discovery: current insights. *Adv. Genomics Genet.* **7,** 29–45 (2017).

37. Edwards, R. A., McNair, K., Faust, K., Raes, J. & Dutilh, B. E. Computational approaches to predict bacteriophage-host relationships. *FEMS Microbiol. Rev.* **40,** 258–272 (2016).

38. Roux, S., Hallam, S. J., Woyke, T. & Sullivan, M. B. Viral dark matter and virus-host interactions resolved from publicly available microbial genomes. *Elife* **4,** e08490 (2015).

39. Ahlgren, N., Ren, J., Lu, Y. Y., Fuhrman, J. A. & Sun, F. Alignment-free d_2^* oligonucleotide frequency dissimilarity measure improves prediction of hosts from metagenomically-derived viral sequences. *Nucleic Acids Res.* **45,** 39–53 (2016).

40. Galiez, C., Siebert, M., Enault, F., Vincent, J. & Söding, J. WIsH: who is the host? Predicting prokaryotic hosts from metagenomic phage contigs. *Bioinformatics* **33,** 3113–14 (2017).

41. Mihara, T. *et al.* Linking Virus Genomes with Host Taxonomy. *Viruses* **8,** 66 (2016).

42. Villarroel, J. *et al.* HostPhinder: A phage host prediction tool. *Viruses* **8,** 116 (2016).
